# RUFY4 deletion prevents pathological bone loss by blocking endo-lysosomal trafficking of osteoclasts

**DOI:** 10.1038/s41413-024-00326-8

**Published:** 2024-05-15

**Authors:** Minhee Kim, Jin Hee Park, Miyeon Go, Nawon Lee, Jeongin Seo, Hana Lee, Doyong Kim, Hyunil Ha, Taesoo Kim, Myeong Seon Jeong, Suree Kim, Taesoo Kim, Han Sung Kim, Dongmin Kang, Hyunbo Shim, Soo Young Lee

**Affiliations:** 1 Department of Life Science, Ewha Womans University, Seoul, 03760 South Korea; 2 The Research Center for Cellular Homeostasis, Ewha Womans University, Seoul, 03760 South Korea; 3 Department of Biomedical Engineering, Yonsei University, Wonju, 26493 South Korea; 4 KM Convergence Research Division, Korea Institute of Oriental Medicine, Daejeon, 34054 South Korea; 5 Chuncheon Center, Korea Basic Science Institute, Chuncheon, 24341 South Korea; 6 Fluorescence Core Imaging Center and Bioimaging Data Curation Center, Ewha Womans University, Seoul, 03760 South Korea; 7 Multitasking Macrophage Research Center, Ewha Womans University, Seoul, 03760 South Korea

**Keywords:** Bone, Pathogenesis

## Abstract

Mature osteoclasts degrade bone matrix by exocytosis of active proteases from secretory lysosomes through a ruffled border. However, the molecular mechanisms underlying lysosomal trafficking and secretion in osteoclasts remain largely unknown. Here, we show with GeneChip analysis that RUN and FYVE domain-containing protein 4 (RUFY4) is strongly upregulated during osteoclastogenesis. Mice lacking *Rufy4* exhibited a high trabecular bone mass phenotype with abnormalities in osteoclast function in vivo. Furthermore, deleting *Rufy4* did not affect osteoclast differentiation, but inhibited bone-resorbing activity due to disruption in the acidic maturation of secondary lysosomes, their trafficking to the membrane, and their secretion of cathepsin K into the extracellular space. Mechanistically, RUFY4 promotes late endosome-lysosome fusion by acting as an adaptor protein between Rab7 on late endosomes and LAMP2 on primary lysosomes. Consequently, *Rufy4*-deficient mice were highly protected from lipopolysaccharide- and ovariectomy-induced bone loss. Thus, RUFY4 plays as a new regulator in osteoclast activity by mediating endo-lysosomal trafficking and have a potential to be specific target for therapies against bone-loss diseases such as osteoporosis.

## Introduction

Bone remodeling is a dynamic process that balances calcified bone matrix resorption by osteoclasts against new bone matrix formation by osteoblasts. The coupling of bone resorption to formation is tightly regulated by communications between osteoclasts and osteoblasts. An imbalance in remodeling can result in metabolic bone diseases such as osteoporosis and osteopetrosis.^1,2^ Therefore, to better understand the pathogenesis of bone diseases, it is essential to identify the molecular mechanisms that underlie osteoclast activity. This will also aid the discovery of new targets with therapeutic potential.

Osteoclasts are large, multinucleated cells derived from hematopoietic stem cells.^3^ Upon maturation and attachment to bone surface, osteoclasts form a sealing zone, which is an actin-rich ring-like structure that surrounds a ruffled border. Beneath the ruffled border, hydrolases from lysosomal vesicles, including cathepsin K (CTSK), are secreted into the extracellular space to degrade the bone matrix.^4–6^ Thus, the biogenesis, trafficking, and exocytosis of lysosomes in osteoclasts must be tightly controlled for proper bone resorption. However, the precise mechanisms by which lysosomes are regulated in osteoclasts remain poorly understood.

In general, lysosomes are intracellular digestive organelles that are considered to be the end-point of endocytosis and autophagy.^7^ Several studies have shown that various cellular stimuli can induce lysosomes to undergo exocytosis, which involves the fusion of lysosomes with the plasma membrane and the discharge of their contents into the extracellular space.^8,9^ Lysosomal exocytosis is a fundamental process that plays many important roles in the body: for example, it mediates antigen presentation by antigen-presenting cells,^10^ neurotransmitter release by neurons,^11^ and, as mentioned above, bone resorption by osteoclasts.^12^ Lysosomal exocytosis involves the rapid exchange of membrane components and cargo between early/late endosomes and primary lysosomes that generates transient hybrid organelles called endo-lysosomes; these then develop into secondary lysosomes.^13–16^ The fusion of the late endosomes with primary lysosomes involves proteins such as the multi-subunit homotypic fusion and vacuole protein sorting (HOPS) tethering complex: this binds small membrane-associated GTPases such as Rab7 and assembles SNARE proteins.^17–19^

The RUN and FYVE domain-containing (RUFY) protein family consists of five adaptor proteins that participate in intracellular trafficking and cytoskeletal dynamics. The proteins are called RUFY1 to RUFY4 and FYCO1, which is a RUFY4 paralogue. All share a common structure, including an N-terminal RUN domain that interacts with small GTPases, one or more coiled-coil (CC) repeats, and a phosphatidylinositol 3-phosphate (PI(3)P)-interacting C-terminal FYVE domain.^20,21^ Recent studies have shown that RUFY4 is an effector of small GTPases such as Rab7^22^ and ARL8.^23^ Notably, Rab7 is highly expressed at the ruffled border of bone-resorbing osteoclasts and its knockdown disrupts both the targeting of vesicles to the ruffled border and bone resorption.^24^ Given these facts, RUFY4 may contribute to osteoclast function by mediating endosome-lysosome fusion. However, to date, no studies on the role of RUFY4 in bone have been conducted.

In this study, we generated whole-body *Rufy4* knockout mice to determine the role of RUFY4 in osteoclastogenesis. These mice exhibited increased trabecular bone mass due to impaired bone resorption, and this associated with reduced lysosomal maturation, lysosome trafficking to the membrane, ruffled border formation, and CTSK secretion. Mechanistic studies revealed that RUFY4 functions as an adaptor protein that enables the interaction between Rab7 on late endosomes and LAMP2 on primary lysosomes. Furthermore, *Rufy4* deletion protected mice from lipopolysaccharide (LPS)- and ovariectomy (OVX)-induced bone loss. Thus, our results demonstrated that RUFY4 positively regulates osteoclast function by mediating endo-lysosomal trafficking and could be a therapeutic target for bone diseases.

## Results

### *Rufy4* deficiency increases trabecular bone mass in mice

To identify genes whose mRNA expression levels change significantly during osteoclastogenesis, bone marrow-derived macrophages (BMMs) from wild-type mice were treated with or without RANKL and subjected to GeneChip analysis. This showed that osteoclastogenesis associated with *Rufy4*, which was 22.36-fold upregulated at 72 h in a time-dependent manner (Fig. S1a). These findings were confirmed by qRT-PCR analysis: RANKL treatment of the BMMs dramatically increased the expression of *Rufy4* mRNA (Fig. S1b). Interestingly, the upregulation of *Rufy4* expression was much greater than that of other *Rufy* family members (Fig. S1c). Considering that nuclear factor of activated T cells cytoplasmic 1 (NFATc1) is a master transcription factor for osteoclast differentiation^25^ and nuclear factor kappa B p65 (NF-κB p65) is an essential transcription factor that is upregulated by RANKL,^26^ we asked whether they were involved in *Rufy4* expression. Indeed, both cyclosporin A, a known inhibitor of NFATc1, and BAY11-7082, an NF-κB inhibitor, suppressed RANKL-induced *Rufy4* expression (Fig. S1d, e). Furthermore, luciferase reporter assays showed that two of three putative NFATc1-binding sites and the majority of the predicted NF-κB p65-binding sites in the *Rufy4* promoter exhibited high luciferase activity (Fig. S1f–h). These results show that both NFATc1 and especially NF-κB p65 can mediate *Rufy4* expression. Next, to dissect the role of RUFY4 in osteoclasts, we overexpressed RUFY4 in BMMs via retroviral transduction. Overexpression of RUFY4 did not affect osteoclast differentiation (Fig. S2a–c). However, RUFY4 overexpression significantly increased bone resorption as measured with hematoxylin staining (Fig. S2d). These data indicate that RANKL-induced *Rufy4* expression is involved in osteoclast function, not osteoclast formation.

To further characterize the physiological roles of RUFY4, we generated *Rufy4* global knockout mice (Fig. S3a–c). Since loss of *Rufy4* did not affect body weight (Fig. S3d), it seems that RUFY4 function is independent of normal development and whole-body growth. Moreover, *Rufy4* deficiency did not alter the expression of any other *Rufy* family member in osteoclasts (Fig. S3e). Terawaki et al.^22^ previously showed that *Rufy4* positively regulates IL-4-induced autophagic flux in dendritic cells (DCs). However, we found that deleting *Rufy4* did not affect the bone marrow-derived DCs (BMDCs) population regardless of whether they were stimulated with or without LPS (Fig. S4a, b), nor did it affect the *CD86* expression in differentiated BMDCs (Fig. S4c). Further, autophagy-related signaling was unchanged in *Rufy4*^*−/−*^ osteoclasts (Fig. S4d). This discrepancy could be caused by functional differences between the cell types.

**Fig. 1:**
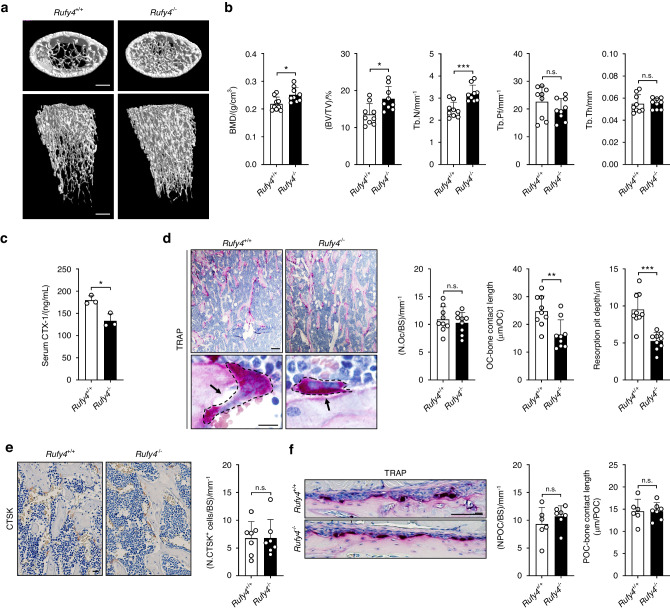
*Rufy4* deficiency increases trabecular bone mass in mice. **a** Representative micro‑computed tomography (micro-CT) images of the distal femur from 8-week-old male *Rufy4*^*+/+*^ and *Rufy4*^*−/−*^ mice. Scale bars, 500 μm. **b** Quantification of BMD, BV/TV, Tb.N, Tb.Pf, and Tb.Th in the distal femur and proximal tibia (*n* = 9 mice per group). **c** CTX-1 protein levels in the serum (*n* = 3 mice per group). **d** Representative TRAP staining images of the femurs in (**a**). The dashed lines indicate osteoclasts. Black arrows indicate the bone areas that were resorbed by the osteoclasts. Scale bars, 100 μm (top) and 10 μm (bottom). Quantification of osteoclast number per bone surface (N.OC/BS), length of osteoclasts contact with bone, and resorption pit depth. **e** Representative images of the femurs in (**a**) after immunohistochemical staining for CTSK. Quantification of CTSK-expressing cell number per bone surface (N.CTSK^+^ cells/BS) (*n* = 7 mice per group). **f** Representative images of the periosteal bone surface after TRAP staining of the femurs in (**a**). Quantification of periosteal osteoclast number per bone surface (N.POC/BS) and length of periosteal osteoclasts contact with bone (*n* = 6–7 mice per group). Scale bar, 100 μm. The data are presented as mean ± SD. Two-tailed unpaired Student’s *t* test was used for statistical analysis. *P < 0.05; **P < 0.01; ***P < 0.001; n.s. not significant

Next, we used *Rufy4*^*+/+*^ and *Rufy4*^*−/−*^ mice to study the effects of *Rufy4* deficiency on bone homeostasis. Surprisingly, at the age of 2 months, *Rufy4*-deficient mice exhibited higher trabecular bone mass compared to control mice (Fig. 1a). However, no differences were found in terms of cortical bone phenotype (Fig. S5). *Rufy4*^*−/−*^ mice also had significantly higher trabecular bone mineral density (BMD) and bone volume fraction (BV/TV) that was mainly due to increased trabecular numbers (Tb.N) rather than changes in the pattern (Tb.Pf) or thickness of the individual trabeculae (Tb.Th) (Fig. 1b). These changes in bone phenotype were still observed in older mice (Fig. S6). In addition, *Rufy4*^*−/−*^ mice had significantly lower serum levels of c-telopeptide of type I collagen (CTX-1), a bone resorption marker (Fig. 1c). Notably, although the bones from *Rufy4*^*+/+*^ and *Rufy4*^*−/−*^ mice had similar numbers of tartrate-resistant acid phosphatase (TRAP)^+^ or CTSK^+^ osteoclasts, *Rufy4*^*−/−*^ osteoclasts were more rounded and the length of their contact with the bone was much shorter. Consequently, the resorption pit depth was also much smaller (Fig. 1d, e). However, periosteal osteoclasts, which are mostly unfused mononuclear cells,^27^ did not show any differences in *Rufy4*^*−/−*^ mice (Fig. 1f). These data suggest that the high trabecular bone mass observed in *Rufy4* knockout mice is due to abnormalities in osteoclast bone resorption rather than osteoclast formation.

### RUFY4 promotes bone resorption by mediating the movement of lysosomes toward the ruffled border

**Fig. 2:**
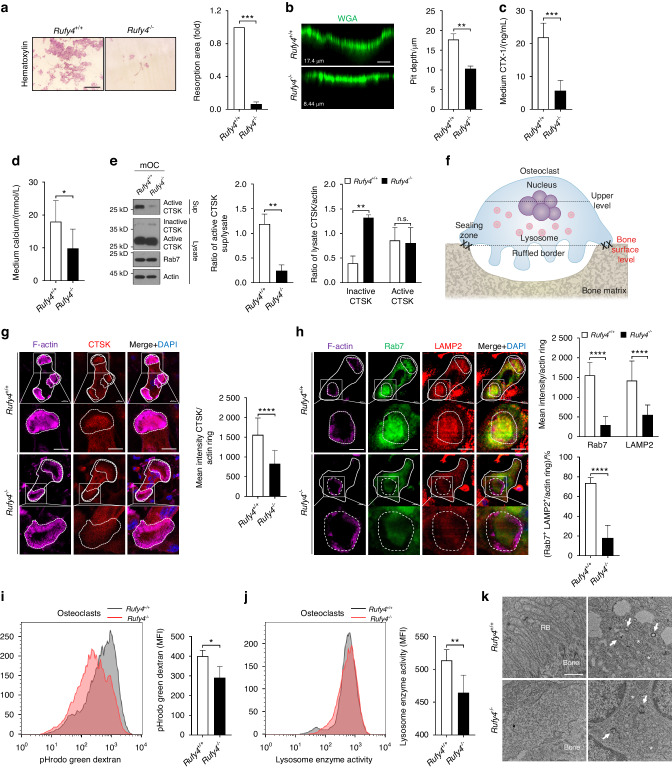
Loss of *Rufy4* impairs osteoclast function due to defective lysosome distribution. **a**–**h** BMMs from 6–8-week-old male *Rufy4*^+/+^ and *Rufy4*^−/−^ mice were cultured with M-CSF and RANKL on bone slices. **a**, **b** Hematoxylin (**a**) or WGA (**b**) staining was conducted to visualize the resorption pit area and depth, respectively (*n* = 3). Scale bars, 250 μm (**a**) and 10 μm (**b**). **c** CTX-1 protein levels in the culture medium (*n* = 8). **d** Calcium levels in the culture medium (*n* = 6). **e** Immunoblot analysis of active and inactive CTSK levels in the cell lysates and supernatants (*n* = 3). Actin served as a loading control. Western blots were probed with the indicated antibodies. Band intensity was determined with ImageJ. **f** Schematic depiction of a mature osteoclast cultured on bone. **g**, **h** Immunofluorescence staining of CTSK (**g**) or Rab7 and LAMP2 (**h**) in osteoclasts at the bone surface level as described above in (**f**). Mean intensity of each protein per actin ring or the percentage of both Rab7^+^ and LAMP2^+^ in actin ring was quantified (*n* = 3). The dashed lines indicate the actin rings in osteoclasts. Scale bars, 10 μm. **i**, **j** BMMs from 6–8-week-old male *Rufy4*^+/+^ and *Rufy4*^−/−^ mice were cultured with M-CSF and RANKL on a plastic surface. Flow cytometry of pHrodo green dextran (**i**) or lysosome enzyme activity (**j**) was conducted. Mean fluorescence intensity (MFI) of FITC was quantified (*n* = 5). **k** Representative electron microscopic images of *Rufy4*^+/+^ and *Rufy4*^−/−^ osteoclasts cultured on bone slices. White asterisks and white arrows indicate mitochondria and autophagosomes, respectively. Scale bar, 1 μm. WGA wheat germ agglutinin, CTSK cathepsin K, Sup supernatants, RB ruffled border. The data are presented as mean ± SD. Two-tailed unpaired Student’s *t* test was used for statistical analysis. *P < 0.05; **P < 0.01; ***P < 0.001; ****P < 0.000 1; n.s. not significant

To investigate the intracellular mechanisms underlying the high bone mass phenotype in *Rufy4*^*−/−*^ mice, we cultured BMMs from *Rufy4*^*+/+*^ and *Rufy4*^*−/−*^ mice with M-CSF and RANKL in vitro and then conducted the resorption pit formation assay using hematoxylin or wheat germ agglutinin (WGA), CTX-1 ELISA assay, and calcium colorimetric assay. Consistent with the in vivo results, *Rufy4* deficiency markedly reduced the bone resorption activity of osteoclasts (Fig. 2a–d) but did not affect the differentiation of osteoclasts (Fig. S7). Since inhibiting osteoclast-mediated bone resorption could affect osteoblast-mediated bone formation due to coupling of two processes,^28^ we analyzed the osteoblast activity in *Rufy4*^*+/+*^ and *Rufy4*^*−/−*^ mice. The results showed that *Rufy4* deficiency did not significantly alter any bone formation indicators, namely the mineral apposition rate, mineralizing surface, bone formation rate, osteoblast number, or serum levels of procollagen type I N-terminal propeptide (PINP), a bone formation marker (Fig. S8a–d). Moreover, *Rufy4*-deficient bone marrow stromal cells (BMSCs) formed comparable numbers of CFU-F colonies compared to control cells (Fig. S8e). In addition, when BMSCs or calvarial osteoprogenitors from both groups were differentiated into osteoblasts, their osteogenic differentiation was similar, as shown by alkaline phosphatase (ALP) activity, the mineralization revealed by Alizarin Red S (ARS), and the expression of osteoblast-specific genes (Fig. S8f–i).

Upon adhesion to bone matrix, osteoclasts reorganize their cytoskeleton to form stable actin rings and then secrete lysosomal proteases into the extracellular space.^5,29,30^ Immunofluorescence and immunoblot analyses of mature osteoclasts showed that *Rufy4*^*−/−*^ osteoclasts have well-organized actin rings and acetylated tubulin (Fig. S9). Therefore, we next considered the possibility that RUFY4 may regulate the exocytotic release of CTSK, which is a major hydrolase responsible for bone resorption. Interestingly, immunoblot analysis revealed that mature *Rufy4*^*+/+*^ and *Rufy4*^*−/−*^ osteoclasts on dentin slices had similar amounts of active CTSK in the cell lysate, whereas *Rufy4*^*−/−*^ osteoclasts secreted much less active CTSK into the supernatant and accumulated more inactive CTSK (Fig. 2e). This pattern was also observed when osteoclasts were cultured on a plastic surface (Fig. S7b). In addition, as shown by immunofluorescence analysis, total CTSK protein expression did not change (Fig. S10a), but *Rufy4*^*−/−*^ osteoclasts had less CTSK inside the actin rings at the bone surface (Fig. 2f, g). These data suggest that RUFY4 plays a pivotal role in the activation and secretion of CTSK.

Given that RUFY4 interacts with Rab7, an essential regulator of endo-lysosomal vesicular trafficking,^22,31^ we hypothesized that the disrupted CTSK secretion in *Rufy4*^*−/−*^ osteoclasts may be due to defective endo-lysosomal transport to the ruffled border membrane. We thus examined the localization of Rab7 and LAMP2, two common late endosome and lysosome markers,^32,33^ in osteoclasts cultured on bone slices by immunofluorescence analysis. Although *Rufy4* deficiency did not affect the perinuclear levels of Rab7 or LAMP2 (Fig. S10b), *Rufy4*^*−/−*^ osteoclasts had very little Rab7 and LAMP2 inside the actin rings. Interestingly, Rab7 and LAMP2 co-localized closely in *Rufy4*^*+/+*^ osteoclasts but this was greatly disrupted in *Rufy4*^*−/−*^ osteoclasts (Fig. 2h). By contrast, *Rufy4* deficiency had no effect on the localization of early or recycling endosomes (Fig. S10c–f). To determine whether the disturbed endo-lysosome distribution in *Rufy4*^*−/−*^ osteoclasts reduces acidic lysosome maturation, osteoclasts were treated with dextran labeled with pH-sensitive pHrodo green or pH-insensitive Alexa Fluor 546 fluorescent dye. Indeed, flow cytometry showed that while the intracellular Alexa Fluor 546 dextran fluorescence was similar in *Rufy4*^*+/+*^ and *Rufy4*^*−/−*^ osteoclasts (Fig. S10g), *Rufy4* deficiency significantly decreased pHrodo green dextran fluorescence (Fig. 2i). This indicates that *Rufy4* deficiency blocks the late stage of lysosome maturation but does not affect the early endo-lysosome pathway. These findings were also confirmed by the lower lysosomal intracellular activity in *Rufy4*^*−/−*^ osteoclasts (Fig. 2j). Moreover, these changes in lysosomal enzyme activity were not due to impaired lysosome biogenesis since *Rufy4*^*+/+*^ and *Rufy4*^*−/−*^ osteoclasts did not differ in the expression of the transcription factor EB (TFEB) that regulates lysosome biogenesis or its target genes *Scarb2*, *Lamp2*, and *Cd63* (Fig. S10h).^14,34,35^ Considering that lysosomal trafficking is essential for the formation of the ruffled border,^36^ we assessed the effect of *Rufy4* deficiency on ruffled border formation using transmission electron microscopy. Although *Rufy4*^*−/−*^ osteoclasts had an intact sealing zone, they lacked ruffled borders (Fig. 2k). These data suggest that RUFY4 promotes lysosomal maturation and its transport to the peripheral ruffled border.

### Expression of WT RUFY4 rescues the phenotypes of *Rufy4*^*−/−*^ osteoclasts

**Fig. 3:**
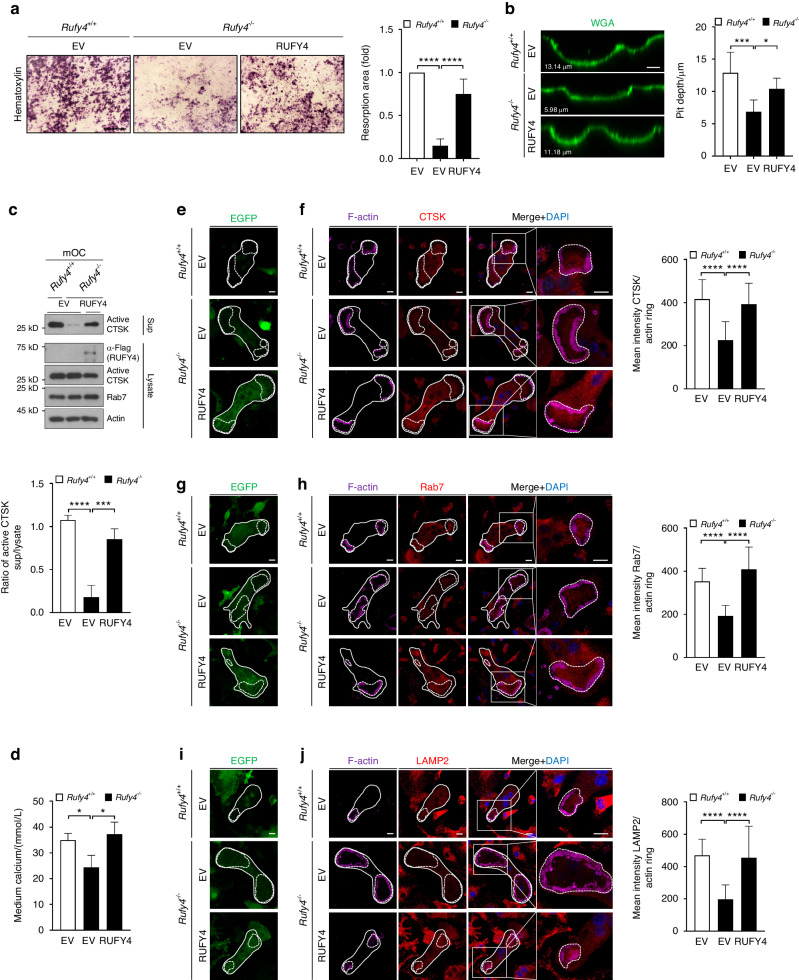
Transduction of RUFY4 rescues the bone-resorbing activity in *Rufy4*^−/−^ osteoclasts. BMMs from 6–8-week-old male *Rufy4*^+/+^ and *Rufy4*^−/−^ mice were transduced with empty vector (EV) or RUFY4 prior to culture on bone slices. **a**, **b** Hematoxylin (**a**) or WGA (**b**) staining were conducted to visualize the resorption pit area and depth, respectively (*n* = 6). Scale bars, 250 μm (**a**) and 10 μm (**b**). **c** Immunoblot analysis of active CTSK levels in the cell lysates and supernatants (*n* = 3). Actin served as a loading control. Western blots were probed with the indicated antibodies. Band intensity was determined with ImageJ. **d** Calcium levels in the culture medium (*n* = 3). **e**–**j** Immunofluorescence staining of CTSK (**e**, **f**), Rab7 (**g**, **h**), and LAMP2 (**i**, **j**) in osteoclasts at the bone surface level (*n* = 3). The EGFP signal from the transduced EV or RUFY4 are shown. Mean intensity of each protein per actin ring was quantified (*n* = 3). The dashed lines indicate the actin rings in osteoclasts. Scale bars, 10 μm. The data are presented as mean ± SD. Two-way ANOVA with Tukey’s multiple comparison post hoc test was used for statistical analysis. *P < 0.05; ***P < 0.001; ****P < 0.000 1

We next tested whether restoring wild-type RUFY4 could rescue the reduction in osteoclast activity caused by *Rufy4* gene deletion. When *Rufy4*^*−/−*^ BMMs were transduced with a full length RUFY4-expressing retroviral vector, osteoclastic bone resorption was significantly restored, as demonstrated by pit formation, CTSK secretion, and calcium release (Fig. 3a–d). In addition, immunofluorescence analysis showed that adding EGFP-tagged RUFY4 rescued the CTSK and lysosome distribution inside the actin rings (Fig. 3e–j). These data suggest that the phenotypes of *Rufy4*^*−/−*^ osteoclasts can be attributed entirely to the *Rufy4* deficiency.

### RUFY4 interacts with Rab7 and LAMP2 to regulate late endosome-lysosome fusion

**Fig. 4:**
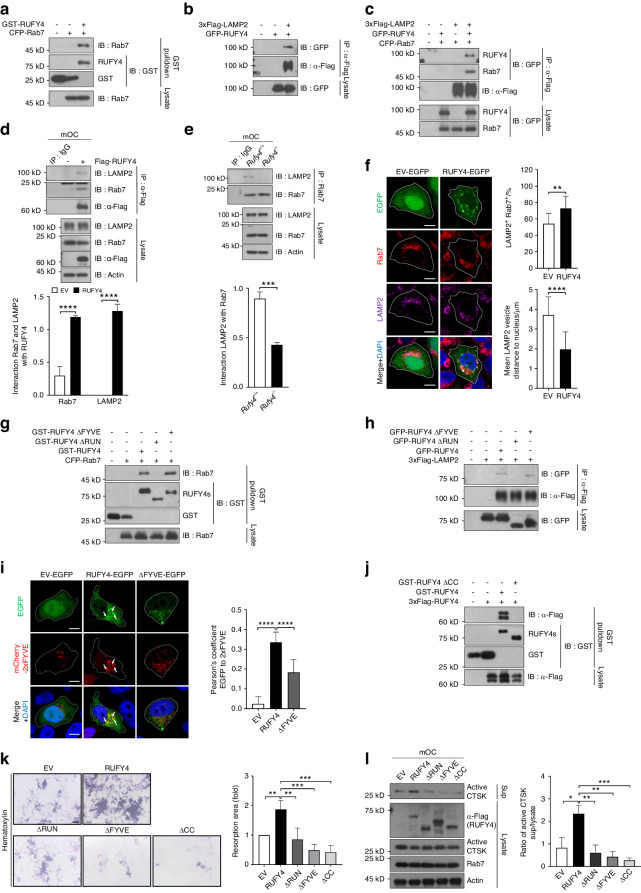
RUFY4 acts as an adaptor between Rab7 and LAMP2. **a**–**c** Co-IP analysis of association of RUFY4 with either Rab7 (**a**), LAMP2 (**b**), or both (**c**) in HEK293T cells that were transfected to express the indicated proteins (*n* = 3). **d** Co-IP analysis of endogenous association of Rab7 and LAMP2 with transduced Flag-RUFY4 in mature osteoclasts (*n* = 3). **e** Co-IP analysis of endogenous association of Rab7 and LAMP2 in mature *Rufy4*^+/+^ and *Rufy4*^−/−^ osteoclasts (*n* = 3). **f** Immunofluorescence staining of endogenous Rab7 and LAMP2 in HeLa cells that were transfected with EV-EGFP or RUFY4-EGFP. In EGFP-expressing cells, the percentage of Rab7^+^ LAMP2^+^ area among total vesicles or mean LAMP2 vesicle distance to nucleus was quantified (*n* = 3). **g**, **h** Co-IP analysis of association between ΔRUN or ΔFYVE mutants of RUFY4 with Rab7 (**g**) or LAMP2 (**h**) in HEK293T cells (*n* = 3). **i** Fluorescence staining of HeLa cells that were co-transfected with EV-EGFP, RUFY4-EGFP, or ΔFYVE-EGFP, and mCherry-2xFYVE. White arrows indicate the co-localization of RUFY4-EGFP with mCherry-2xFYVE, which labels PI(3)P-containing organelles (*n* = 3). Scale bars, 10 μm. **j** Co-IP analysis of association between ΔCC mutant of RUFY4 and full-length RUFY4 in HEK293T cells (*n* = 3). **k**, **l** *Rufy4*^−/−^ osteoclasts were transduced with full-length RUFY4 or its domain mutants and cultured on bone slices. **k** Hematoxylin staining was conducted to visualize resorption pits (*n* = 5). Scale bar, 100 μm. **l** Immunoblot analysis of active CTSK levels in the cell lysates and supernatants (*n* = 3). Actin served as a loading control. Western blots were probed with the indicated antibodies. Band intensity was determined with ImageJ. The data are presented as mean ± SD. Two-tailed unpaired Student’s *t* test (**d**–**f**) or one-way ANOVA with Tukey’s multiple comparison post hoc test (**i**, **k**, **l**) was used for statistical analysis. *P < 0.05; **P < 0.01; ***P < 0.001; ****P < 0.000 1

Our finding that deleting *Rufy4* affects the endo-lysosomal trafficking in osteoclasts is consistent with Terawaki et al., who showed that RUFY4 mediates autophagosome-endo/lysosome fusion in DCs. They also showed that RUFY4 exerts this function by acting as a Rab7 effector.^22^ Notably, as shown in Fig. 2h, the native co-localization of Rab7 and LAMP2 in *Rufy4*^*+/+*^ osteoclasts was greatly disrupted in *Rufy4*^*−/−*^ osteoclasts. This together with the findings of Terawaki et al.^22^ led us to further elucidate the molecular mechanisms by which RUFY4 regulates the distribution of late endosomes and lysosomes. To address this, we co-expressed RUFY4 with Rab7 or LAMP2 in HEK293T cells and then subjected to co-immunoprecipitation (co-IP) analyses. Indeed, RUFY4 interacts with both Rab7 and LAMP2 (Fig. 4a, b). In addition, immunofluorescence analyses showed that RUFY4-EGFP colocalizes with both Rab7 and LAMP2 in HeLa cells (Fig. S11a, b). Interestingly, Rab7 and LAMP2 interacted only when they were co-expressed with RUFY4 (Fig. 4c). This was also observed in mature osteoclasts: RUFY4 formed a complex with endogenous Rab7 and LAMP2 in *Rufy4*-overexpressing mature wild-type osteoclasts (Fig. 4d), but Rab7 did not bind to LAMP2 in *Rufy4*^*−/−*^ osteoclasts (Fig. 4e). Notably, compared to HeLa cells that overexpressed only EGFP, RUFY4-EGFP-overexpressing HeLa cells also showed an increase in Rab7^+^ LAMP2^+^ endo-lysosome numbers that were more enriched in the juxtanuclear region (Fig. 4f). These results suggest that RUFY4 acts as an adaptor protein between Rab7 and LAMP2.

The RUFY4 protein bears an N-terminal RUN domain, one CC domain, and a C-terminal FYVE domain.^21^ To determine which RUFY4 domain affects its interaction with Rab7 and LAMP2, HEK293T and HeLa cells were induced to overexpress RUFY4 domain deletion mutants (Fig. S11c). Co-IP and immunofluorescence analyses showed that deleting the RUN domain reduced the interaction between Rab7 and RUFY4 (Fig. 4g and Fig. S11d), which is consistent with Terawaki et al.^22^ Deletion of the RUN domain also dramatically decreased the binding of RUFY4 with LAMP2 (Fig. 4h and Fig. S11e). The FYVE domain deletion did not interfere with the RUFY4 interaction with Rab7 and LAMP2 (Fig. 4g, h and Fig. S11d, e). However, the FYVE domain is known to be required for binding to PI(3)P,^22,37^ and indeed, its deletion disrupted the association of RUFY4 with PI(3)P-containing organelles in HeLa cells (Fig. 4i). This suggests that the FYVE domain of RUFY4 mediates endo-lysosomal membrane binding. The CC domain, which is known to be responsible for the dimerization of the RUFY family member FYCO1,^38^ also mediated RUFY4 homo-dimerization (Fig. 4j). In addition, its deletion impaired the interaction between RUFY4 and Rab7 or LAMP2 (Fig. S11d–g). Next, we dissected the role of the three domains of RUFY4 in osteoclast function by transducing *Rufy4*^*−/−*^ BMMs with each deletion mutant. Compared to osteoclasts overexpressing full-length RUFY4, all mutants associated with significantly less bone resorption and CTSK secretion (Fig. 4k, l). However, they did not affect osteoclast differentiation (Fig. S11h). These data together show that the RUN and CC domains of RUFY4 participate in the interaction with Rab7 and LAMP2; moreover, the FYVE domain is needed for endo-lysosomal membrane binding. Thus, all three domains promote endo-lysosome formation, thereby playing critical roles in osteoclast function.

### *Rufy4* deletion ameliorates pathological bone loss

**Fig. 5:**
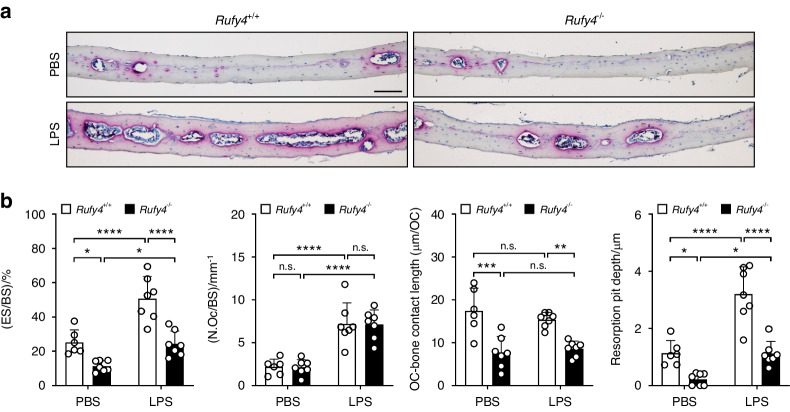
*Rufy4* knockout inhibits LPS-induced bone loss. **a** Representative TRAP staining images of the calvaria from 5–6-week-old male *Rufy4*^+/+^ and *Rufy4*^−/−^ mice that had been injected subcutaneously with PBS or LPS. Scale bar, 100 μm. **b** Quantification of the bone erosion (ES/BS), osteoclast number per bone surface (N.OC/BS), length of osteoclasts contact with bone, and resorption pit depth (*n* = 6–7 mice per group). The data are presented as mean ± SD. Two-way ANOVA with Tukey’s multiple comparison post hoc test was used for statistical analysis. *P < 0.05; **P < 0.01; ***P < 0.001; ****P < 0.000 1; n.s. not significant

Given that RUFY4 promotes osteoclastic bone resorption, we investigated its involvement in bone diseases that are caused by excessive osteoclast activity. For this, we used the LPS and OVX mouse models, which are characterized by pathological bone loss. In the first model, subcutaneous injection of LPS into the calvaria induces inflammatory bone destruction. Indeed, *Rufy4*^*+/+*^ mice demonstrated severe osteolysis in the calvaria. By contrast, *Rufy4*^−/−^ mice displayed mild bone erosion that was similar to that seen in PBS-injected *Rufy4*^+/+^ mice (Fig. 5a, b). While the LPS injection increased osteoclast numbers to similar degrees in *Rufy4*^*+/+*^ and *Rufy4*^−/−^ mice, the osteoclasts in *Rufy4*^−/−^ mice had shorter contact lengths with bone and shallower resorption pits (Fig. 5b), which is consistent with our earlier observations (Fig. 1d).

**Fig. 6:**
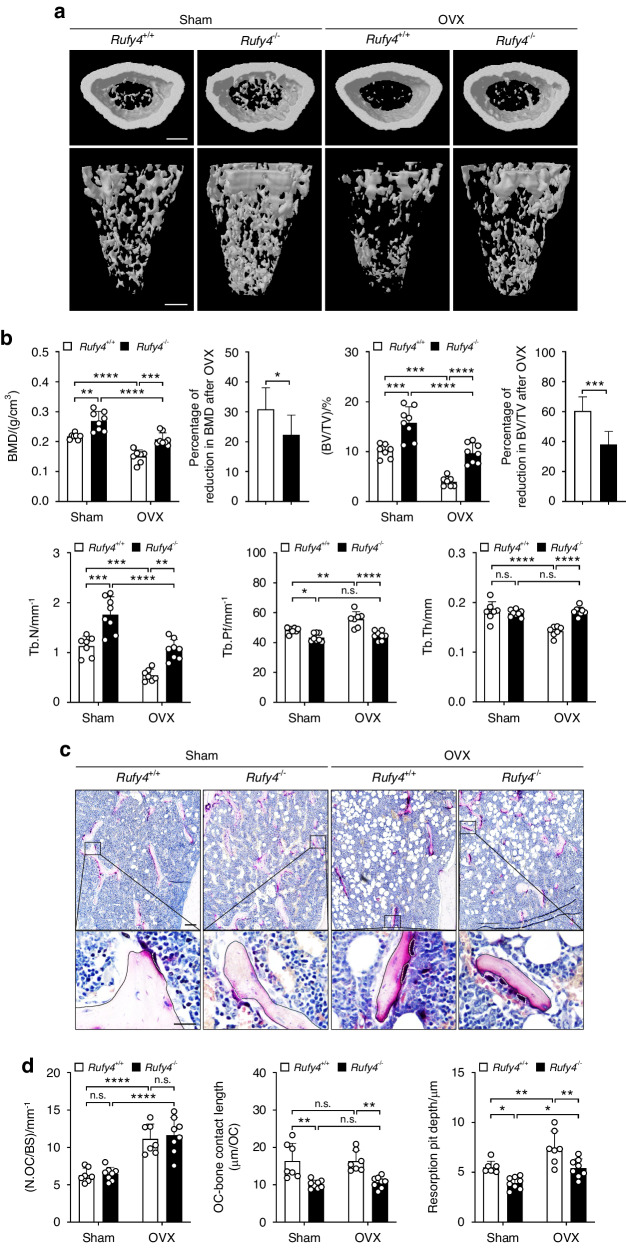
*Rufy4* knockout protects against OVX-induced bone loss. **a** Representative micro-CT images of the distal femur from 10–11-week-old female *Rufy4*^+/+^ and *Rufy4*^−/−^ mice that had been subjected to sham or OVX surgery. Scale bars, 500 μm. **b** Quantification of BMD, the percentage of BMD reduction after OVX relative to sham-operated mice of the same genotype, BV/TV, percentage of BV/TV reduction after OVX relative to sham-operated mice of the same genotype, Tb.N, Tb.Pf, and Tb.Th (*n* = 7–8 mice per group). **c** Representative TRAP staining images of the femurs from the four groups. The white dashed lines indicate osteoclasts. Scale bars, 100 μm (top) and 10 μm (bottom). **d** Quantification of osteoclast number per bone surface (N.OC/BS), length of osteoclasts contact with bone, and resorption pit depth (*n* = 7–8 mice per group). The data are presented as mean ± SD. Two-way ANOVA with Tukey’s multiple comparison post hoc test was used for statistical analysis. *P < 0.05; **P < 0.01; ***P < 0.001; ****P < 0.000 1; n.s. not significant

In the second model, OVX surgery in female mice mimics postmenopausal osteoporosis. As expected, OVX in *Rufy4*^*+/+*^ mice caused significant loss of trabecular bone (Fig. 6a). By contrast, *Rufy4*^−/−^ mice displayed significant preservation of BMD, BV/TV, and Tb.N and unchanged Tb.Pf and Tb.Th (Fig. 6b). Histological analysis revealed that both *Rufy4*^*+/+*^ and *Rufy4*^−/−^ mice had a similar increase in the number of TRAP^+^ osteoclasts, which occurs in response to OVX. However, *Rufy4* deletion reduced the length with which the osteoclasts contacted the bone and their bone resorption activity (Fig. 6c, d). Taken together, these data suggest that *Rufy4* deficiency protects against pathological bone loss in vivo.

## Discussion

**Fig. 7:**
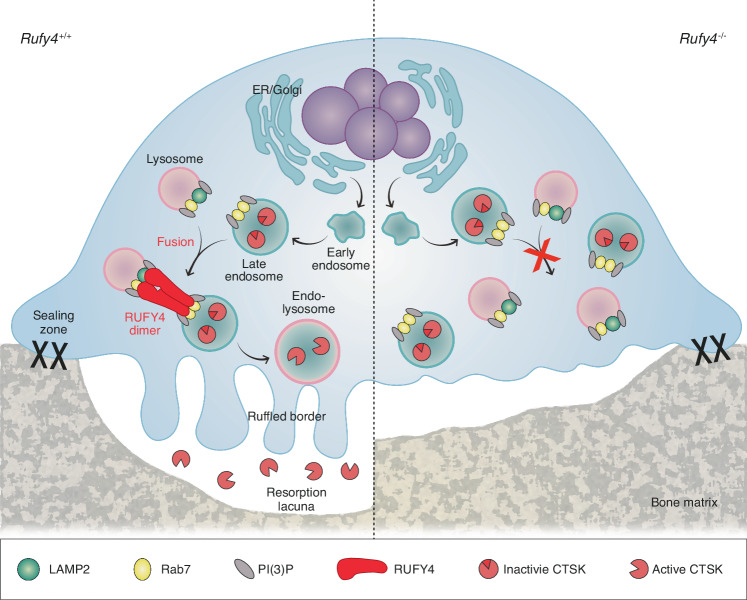
Schematic illustration of the mechanism by which RUFY4-induced endo-lysosomal trafficking promotes bone resorption in osteoclasts. The RUFY4 dimer promotes the fusion of late endosomes and lysosomes by interacting with Rab7 and LAMP2. The endo-lysosomes then move to the ruffled border and undergo exocytosis. This induces the secretion of lysosomal hydrolases such as active CTSK into the extracellular space near the bone. Consequently, resorption lacunae emerge. CTSK cathepsin K

During bone resorption, osteoclasts secrete lysosomal hydrolases such as CTSK into the resorption lacunae.^4–6^ However, the mechanisms that control the trafficking of hydrolase-containing lysosomes to the ruffled border are not well understood. Here, our study on RUFY4 provided insights into how endo-lysosomal trafficking occurs in osteoclasts (Fig. 7). Specifically, we observed that knocking out *Rufy4* in mice reduced osteoclast resorption activity without affecting osteoclast or osteoblast differentiation (Fig. 2a–d and Fig. S7–8). Surprisingly, this reduction was not due to dysfunction in actin ring formation, early or recycling endosome localization, endocytosis, or lysosome biogenesis (Figs. S9 and S10). Rather, it associated with acidic maturation of the lysosome and impaired movement of this organelle to the membrane. This impeded lysosome exocytosis, which in turn prevented the formation of the ruffled border and blocked the lysosomal secretion of CTSK into the extracellular space (Fig. 2e–k). Mechanistic studies revealed that RUFY4 mediates late endosome-lysosome fusion by acting as a bridge between Rab7 on late endosomes and LAMP2 on primary lysosomes (Fig. 4a–f and Fig. S11a, b). We also found that this bridging activity of RUFY4 is mediated by all of three domains in RUFY4: the RUN domain enables the binding of RUFY4 to Rab7 and LAMP2; the FYVE domain mediates the binding of RUFY4 to the PI(3)P proteins on both late endosomes and primary lysosomes; and the CC domain regulates the homo-dimerization of RUFY4 that is needed for it to bind to Rab7 and LAMP2 (Fig. 4g–l and Fig. S11c–g). Most importantly, we showed the increased trabecular bone mass in *Rufy4*^−/−^ mice under both physiological and pathological conditions (Figs. 1, 5 and 6 and Fig. S6a).

Lysosomal cysteine proteases, including CTSK, are activated and functional at acidic pH.^39^ Active CTSK is essential for bone collagen degradation in osteoclasts,^40^ so its activation is crucial. We showed that the secretion of active CTSK is disturbed in mice lacking *Rufy4*. While *Rufy4*^+/+^ and *Rufy4*^−/−^ osteoclasts expressed CTSK mRNA and total protein at similar levels, the mutant lysates had significantly greater amounts of inactive CTSK (Fig. 2e, Fig. S7b, c and Fig. S10a). These results suggest that RUFY4 promotes CTSK activation and secretion, but not its expression.

Several other proteins have been shown to participate in endosome-lysosome fusion and the maturation and trafficking of secondary lysosomes in osteoclasts. For example, SNX10 promotes early to late endosome maturation by associating with PIKfyve and EEA1 and plays a crucial role in osteoclast endosomal trafficking, TRAP exocytosis, and bone resorption.^41,42^ Conversely, gasdermin D is localized in the early endosomes and negatively regulates endo-lysosomal trafficking and maturation in osteoclasts by controlling phosphoinositide conversion. Mice lacking gasdermin D show excessive lysosomal activity and bone resorption.^43^ We add to this literature by showing that RUFY4 is critical for the final step of endo-lysosomal maturation and the delivery of lysosomal hydrolases to the ruffled border in osteoclasts.

We noted that *Rufy4* deletion in mice did not fully prevent bone loss induced by LPS or OVX (Figs. 5 and 6). This might be due to a compensatory response to *Rufy4* deficiency. RUFY proteins are structurally similar but functionally different due to unique interaction with Rab GTPase proteins.^21,44^ For example, RUFY1 is involved in early endosome function through its interactions with Rab4, Rab5, and Rab14,^45–47^ and RUFY2 plays a role in autophagosome formation via Rab33A/B interaction.^48,49^ In addition, FYCO1, the RUFY4 paralog, are involved in the fusion of autophagic and endosomal vesicles with lysosomes by interacting with the CCZ1-MON1A complex, which is crucial for Rab7 activation.^50^ Indeed, some of these Rab proteins are expressed in osteoclasts.^51–53^ Furthermore, our study showed that although *Rufy4* was specifically upregulated by RANKL stimulation, all other *Rufy* family members were also slightly induced, regardless of whether *Rufy4* was deleted (Fig. S1c and Fig. S3e), suggesting that other RUFY proteins besides RUFY4 may also contribute to osteoclastogenesis. Thus, compensatory mechanisms by other *Rufy* family members may explain the incomplete protection against pathological bone loss in *Rufy4*^−/−^ mice.

A previous study has revealed that autophagy-related proteins play non-autophagic roles in osteoclast differentiation and function.^54^ DeSelm et al. reported that deficiency in *Atg5* or *Atg7* impairs lysosomal fusion with the membrane and blocks the ruffled border formation, resulting in reduced the bone-resorbing activity of osteoclasts. Similarly, RUFY4 was recently shown to positively regulate autophagy in DCs,^22^ but deleting *Rufy4* did not affect autophagy signaling in osteoclasts (Fig. S4d). These results suggest that the role of RUFY4 in osteoclasts may be independent of the autophagic process.

Excessive osteoclast activity can lead to various bone diseases such as Paget’s disease, tumor-induced osteolysis, and osteoporosis.^55,56^ Current osteoporosis therapies aim to reduce fractures by decreasing bone resorption or increasing bone formation.^57,58^ However, long-term treatment faces challenges, including causing a poor bone quality due to disrupted osteoclast-osteoblast communication.^59,60^ Indeed, bisphosphonates used as antiresorptive drugs have the adverse effect of decreasing bone formation.^61^ Thus, optimal therapies for osteoporosis should keep the osteoclast-osteoblast communication intact. Importantly, we demonstrated that RUFY4 is only specifically involved in the final step of bone resorption, and does not affect osteoclast and osteoblast formation, or osteoblast activity (Fig. 2 and Figs. S7 and S8). These results suggest that RUFY4 can be used as a therapeutic target for the development of drugs that do not cause side effects. Since adaptor proteins such as RUFY4 lack enzymatic activity,^62^ instead of screening for small compounds, research on inhibitory peptides of RUFY4 could lead to promising approaches for future bone therapy.

Our study has several limitations. First, although *Rufy4* deletion in mice was confirmed by PCR and qRT-PCR, we were not able to show whether it reduces RUFY4 protein levels. We were also unable to determine the intracellular distribution of endogenous RUFY4 in osteoclasts. This reflects the lack of commercially available antibodies against RUFY4. To address these issues, we are currently generating our own antibody against RUFY4. Second, while *Rufy4* deletion increased trabecular bone mass and decreased osteoclast activity in mice, no change in cortical bone were detected (Figs. 1 and 2 and Figs. S5 and S6). This may reflect the possibility that RUFY4 also affects the function of other bone cells, including osteocytes, chondrocytes, and endothelial cells. Indeed, previous studies showed that deleting genes systemically or only in specific cell types can result in disparate cortical bone phenotypes.^63,64^ Thus, further studies with osteoclast-specific *Rufy4* conditional knockout mice are required to explore the cell-intrinsic functions of RUFY4 in osteoclasts. Last, the delivery and secretion of secretory lysosomes during bone resorption depends on the dynein-dynactin motor complex.^65–67^ Since a recent study reported that RUFY4 interacts with ARL8 and the dynein-dynactin complex,^23^ further investigation is needed to elucidate the precise mechanisms by which RUFY4 and the dynein-dynactin complex regulate lysosomal trafficking in osteoclasts.

In conclusion, we identified RUFY4 as a novel factor regulating osteoclast function. *Rufy4* knockout resulted in increased trabecular bone mass under physiological conditions. It also ameliorated pathological bone loss in inflammatory and postmenopausal osteoporosis models. Mechanistically, RUFY4 forms a bridge between Rab7 on late endosomes and LAMP2 on lysosomes, thereby promoting endo-lysosomal trafficking, lysosome maturation, CTSK secretion, and ultimately bone resorption. Thus, our study clearly demonstrates that RUFY4 has a significant effect on bone mass and could be a potential therapeutic target for bone-related diseases.

## Materials and methods

### GeneChip analysis

Total RNA was extracted from BMMs cultured with 30 ng/mL M-CSF and 100 ng/mL RANKL and used for cDNA synthesis by reverse-transcription. Biotinylated cDNA was synthesized with Ambion Illumina RNA amplification kit (Ambion). Microarray was performed according to the Illumina GeneChip manual (outsourced to Macrogen).

### Cell culture

BMMs were obtained from tibial and femoral bone of male mice. Bone marrow cells were cultured in α-MEM (Hyclone, USA) containing 10% FBS (Hyclone). After 1 day, non-adherent cells were cultured with 30 ng/mL M-CSF (R&D Systems, USA) for 3 days, as described previously.^68^ Osteoclasts were generated by culturing BMMs with 30 ng/mL M-CSF and 100 ng/mL RANKL (PeproTech, UK).

Bone marrow derived dendritic cells (BMDCs) were obtained from 6–8-week-old mice. Bone marrow cells were isolated and seeded in DMEM (Hyclone) containing 10% FBS with 20 ng/mL GM-CSF. On days 3 and 5, cells were treated with 20 ng/mL rmIL-4. Then on day 7, cells were harvested after incubation with or without 100 ng/mL LPS for 24 h.

Bone marrow stromal cells (BMSCs) were obtained from 4-week-old mice as described previously.^69^ The tibial and femoral bone were cut into small pieces and incubated for 90 min at 37 °C with shaking in a solution containing collagenase type II (C6885, Sigma-Aldrich, USA). The cells were cultured in a 100 mm culture dish.

Primary osteoblasts were harvested from calvaria as described previously.^70^ The calvariae were digested for 15 min in a solution containing collagenase type II (Sigma-Aldrich) and dispase II (Roche) at 37 °C with shaking. The cells were cultured in a 100 mm culture dish. HEK293T cells and HeLa cells were cultured in DMEM (Hyclone).

### Mice

Whole-body *Rufy4* knockout mice were generated using CRISPR/Cas9 technology (outsourced to Macrogen) and backcrossed to a C57BL/6 J background for more than five generations. C57BL/6 J mice were purchased from Jackson Laboratory. All animal experiments were approved by the Institutional Animal Care and Use Committee (IACUC) of Ewha Laboratory Animal Center.

### Micro‑computed tomography (micro-CT) analysis

The left hind limb was harvested, fixed, and scanned using a micro-CT (Skyscan1176, Bruker microCT, Kontich, Belgium). The scanning parameters were set to a voltage of 75 kV, a scanning current of 333 μA, a resolution of 18 μm, an exposure time of 260 ms, and a rotation step of 0.7 deg. The raw data was converted into 2D cross-sectional imageS using NRecon (Brucker micro-CT, Kontich, ver.1.6.9.3, Belgium). Reconstructed images were geometrically aligned with DataViewer (Brucker micro-CT, Kontich, ver.1.5.1.2, Belgium), after which the structural parameters of the tibial and femoral bone were evaluated by CT Analyzer (CT-AN ver.1.10.9.0, Brucker, Belgium). The structural trabecular bone variables are as follows: bone mineral density (BMD, g/cm^3^), which reflects the extent of bone mineral in the bone tissue; bone volume fraction (BV/TV, %); trabecular number (Tb.N, mm^−1^); trabecular bone pattern factor (Tb.Pf, mm^−1^), which is related to connectivity of trabecular bone; trabecular thickness (Tb.Th, mm). The structural cortical bone variables are as follows: BMD (g/cm^3^); bone volume fraction (BV/TV, %); cross-sectional thickness (Ct.Th, mm); cortical bone area (Ct.Ar, mm^2^); total cross-sectional area inside the periosteal envelope (Tt.Ar, mm^2^).

### Histology and immunohistochemistry

The right hind limb was harvested, fixed in 10% formalin for 3 days and decalcified in 0.5 mol/L EDTA for 10 days. 5 μm-thick paraffin sections were stained with ALP or TRAP (Wako, Japan). For immunohistochemistry, sections were incubated with anti-CTSK antibody (sc-48353, Santa Cruz, USA) overnight. Images were obtained under an Olympus BX51 microscope (Olympus) with Olympus DP72 camera (Olympus). Osteoclastic and osteoblastic variables (N.OC/BS, OC-bone contact length, resorption pit depth, N.OB/BS) were analyzed using ImageJ (NIH, USA).

### Bone resorption pit assay

Pre-osteoclasts were seeded on bone slices and cultured with 30 ng/mL M-CSF and 100 ng/mL RANKL for 3 days. The cells adhering to bone slices were removed by mechanical agitation before staining the bone slices with hematoxylin (Sigma-Aldrich) or 20 μg/mL WGA-Alexa 488 conjugate (W11261, Invitrogen, USA) for 30 min. The resorption pit area was visualized using Olympus CKX53 inverted microscope (Olympus, Japan) with microscope digital camera. The resorbed pit area was quantified with Image-Pro Plus (Media Cybernetics, USA). The resorption pit depth was detected with confocal microscope (LSM 880 with Airyscan, Carl Zeiss, Germany) and measured using IMARIS (Bitplane, Switzerland).

### Enzyme-linked immunosorbent assay (ELISA)

Supernatants or serum were collected and the concentrations of CTX-1 or PINP were detected with CrossLaps for Culture CTX-1 ELISA kit (AC-07F1, IDS, UK), RatLaps CTX-1 EIA (AC-06F1, IDS), or Rat/Mouse PINP EIA (AC-33F1, IDS) according to the manufacturer’s instructions.

### Calcium colorimetric assay

Amounts of calcium released after bone resorption were measured with calcium colorimetric assay kit (ab102505, Abcam, USA) according to the manufacturer’s instructions.

### Immunoblot analysis

Cells were lysed in a RIPA buffer containing 0.5% Na-deoxycholate, 50 mmol/L Tris-Cl (pH 8.0), 150 mmol/L NaCl, 1 mmol/L EDTA, 1% NP40, supplemented with protease inhibitors (1 mmol/L PMSF and 1 μg/mL leupeptin and aprotinin) and phosphatase inhibitors (1 mmol/L NaVO_4_ and 1 mmol/L NaF) after vortexing 5 times for 30 min on ice. After centrifugation at 14 000 r/min for 20 min at 4 °C, the supernatants were boiled in 6X SDS buffer containing 0.6 mol/L DTT. The cell lysates or immunoprecipitated proteins from co-IP experiments were separated by 10% SDS-polyacrylamide gels and transferred electrophoretically onto a polyvinylidene difluoride membrane (Millipore, USA). The membranes were blocked with 5% bovine serum albumin (BSA) in Tris-buffered saline containing 0.1% Tween-20 and then immunoblotted with the indicated primary antibodies (Table S1) and secondary antibodies conjugated to HRP. Proteins were detected using ECL detection kit (Biorad, USA).

### Immunofluorescence staining

Cells on glass coverslips or bone slices were fixed with 4% paraformaldehyde in PBS for 15 min. For F-actin staining, cells were permeabilized with 0.2% Triton X-100 in PBS for 5 min and incubated with fluorescent dye-conjugated phalloidin (Invitrogen) for 30 min. For LAMP2 staining, cells were permeabilized with 0.2% saponin in PBS for 1 h and blocked with 0.05% saponin/1% BSA/PBS for 30 min followed by an incubation with primary antibodies overnight. For other immunofluorescence staining, cells were permeabilized and blocked with 5% BSA/0.3% Triton X-100/PBS for 1 h prior to adding primary antibodies (Table S2) in 1% BSA/0.3% Triton X-100/PBS overnight. Following primary antibody incubation, cells were incubated with the respective fluorescent dye-conjugated secondary antibodies (Invitrogen) for 1 h. Nuclei were counterstained with 4’,6-diamidino-2-phenylindole. Fluorescence images were obtained under confocal microscope and the mean intensity per cell or actin ring was measured using IMARIS.

### Measurement of endo-lysosomal pH

Endo-lysosomal pH in osteoclasts was measured as described previously.^43^ Briefly, osteoclasts were incubated with 20 μg/mL pH-sensitive pHrodo green dextran (Thermo Fisher Scientific, USA) or 20 μg/mL pH-insensitive dextran with Alexa Fluor 546 (Thermo Fisher Scientific) for 3 h at 37 °C. The cells were washed 3 times with cold PBS, detached and collected for FACS analysis. Intracellular dextran fluorescence was measured using BD FACS Calibur (BD Biosciences, USA) and analyzed by FlowJo (TreeStar, USA).

### Lysosomal intracellular activity assay

Lysosomal activity was measured with Lysosomal Intracellular Activity kit (Abcam) according to the manufacturer’s instructions. Briefly, osteoclasts were incubated in fresh medium supplemented with 0.5% FBS and 15 μg/mL self-quenched substrate for 1 h at 37 °C. After incubation, the cells were collected, washed twice with Assay buffer, and resuspended in PBS. The intracellular immunofluorescence was measured with BD FACS Calibur (BD Biosciences) and analyzed by FlowJo (TreeStar).

### Transmission electron microscopy

Osteoclasts cultured on bone slices were fixed with 2% glutaraldehyde and 2% paraformaldehyde in phosphate buffer (pH 7.4) for 1 h at 4 °C. The bone slices were decalcified in 5% EDTA/0.1% glutaraldehyde for 3 days and post-fixed with osmium tetroxide for 40 min at 4 °C. The samples were dehydrated in a graded series of ethanol, treated with graded propylene oxide series, and embedded into Epon. The sections were then cut into ultra-thin 80 nm-thick sections and placed on a copper grid. The samples were stained with uranyl acetate and lead citrate and then observed using transmission electron microscope (JEOL-2100F, USA).

### Flow cytometry analysis

Single-cell suspensions in FACS buffer (PBS containing 2% FBS) were prepared and incubated with anti-mouse CD16/CD32 for blocking non-specific Fc receptors binding. The cells were then stained with primary antibodies (Table S3). After 30 min of incubation on ice in the dark, the cells were washed and resuspended in FACS buffer. Flow cytometry was performed on a BD LSRFortessa.

### Constructs

Full-length *Rufy4* cDNA (NM_001170641.1) was amplified from osteoclasts. After PCR amplification, *Rufy4* and its deletion mutants were subcloned into p3xFlag-CMV-13 (Addgene). 3xFlag-tagged RUFY4 and its mutants were subcloned into pMX-Puro plasmid (provided by T. Kitamura, University of Tokyo). pEGFP-N3 and pEBG vectors were obtained from Addgene. pGL3 luciferase reporter vector was obtained from Promega. CMV-CFP-Rab7 vector was provided by Dongmin Kang (Ewha Womans University). LAMP2 expression vector in the pCMV6 vector was obtained from Origene. Amplified LAMP2 from pCMV6-LAMP2 was subcloned into p3xFlag-CMV-13. The primers used are listed in Table S4.

### Retroviral infection

Platinum-E (Plat-E) packaging cells were transfected with various pMX-puro construct DNAs using PEI transfection reagent (Sigma-Aldrich). BMMs were infected with the retroviruses as previously described.^71^ The pMX-puro vector and Plat-E cells were provided by T. Kitamura (University of Tokyo). After viral infection, BMMs were cultured with 30 ng/mL M-CSF and 2 μg/mL puromycine for 3 days. Puromycine-resistant BMMs were used for further analysis.

### Plasmid transfection

HEK293T cells were transfected with the indicated expression vectors using PEI transfection reagent (Sigma-Aldrich) and then subjected to GST-pull down assays or co-IP.

### GST-pull down and co-IP assays

Cells were lysed on ice in RIPA buffer supplemented with protease inhibitors (1 mmol/L PMSF and 1 μg/mL leupeptin and aprotinin). For GST-pull down assay, cell lysates were pulled down by glutathione-Sepharose 4B (GE Healthcare Life Science, USA) for 4 h. After washing three times with RIPA lysis buffer, the beads were boiled with 2X SDS loading buffer. For co-IP assay, cell lysates were incubated with the indicated primary antibodies overnight and were further incubated with protein A-agarose (Millipore) for 1 h at 4 °C with rotation. After washing three times with lysis buffer, the beads were boiled with 2X SDS loading buffer. Immunoblot analysis was then conducted.

### RNA isolation and quantitative real-time PCR (qRT-PCR)

Total RNA was extracted using TRIzol (Invitrogen) and reverse transcribed with RTase kit (Biofact). Polymerase chain reaction (PCR) amplification was performed with SYBR Green Master kit (Bioline) using ABI PRISM 7300 system (Applied Biosystems). Actin primers were used for normalization. The gene-specific primers are listed in Table S5.

### Transcription factor binding site prediction and luciferase reporter assay

NFATc1 and NF-κB p65 binding site sequences were obtained from JASPAR database (https://jaspar.genereg.net/). The prediction of the NF-κB p65 binding site in *Rufy4* promoter was obtained from ALGGEN-PROMO (http://alggen.lsi.upc.es). Each of the predicted *Rufy4* promoter regions (total 4.2 kb: −3 735 ~ −2 338, −2 337 ~ −1 776, −1 775 ~ −90, −455 ~ +462) was cloned into pGL3-basic luciferase vector. HEK293T cells were seeded on 12-well culture plate and then transfected with 900 ng of the above reporter constructs and 0.2 ng of the Renilla luciferase construct with 100 ng of pcDNA-Nfatc1, pcDNA-p65, or a mock plasmid using PEI transfection reagent (Thermo Fisher Scientific) for 24 h. Transfected cells were harvested for dual-luciferase reporter gene assay kit (Promega) according to the manufacturer’s instructions. Firefly and Renilla luciferase were measured with VICTOR X Light Luminescence Plate Reader (PerkinElmer). The reporter activity of all samples was normalized with Renilla luciferase.

### TRAP staining

BMMs were cultured with 30 ng/mL M-CSF and 100 ng/mL RANKL on 48-well culture plate for 4–5 days. The cells were fixed with 4% paraformaldehyde for 10 min and stained with TRAP (Wako).

### Colony-forming unit fibroblast (CFU-F) assay

300 BMSCs were seeded on 6-well culture plate and cultured for 14 days. The cells were fixed with 4% paraformaldehyde for 10 min and stained with crystal violet solution (V5265, Sigma-Aldrich). Colonies that contained more than 50 cells were counted.

### ALP and ARS staining

BMSCs or calvarial osteoprogenitors were cultured with 50 μg/mL L-ascorbic acid, 10 mmol/L β-glycerophosphate, 10 nmol/L dexamethasone, and 50 ng/mL rhBMP2 on 48-well culture plate for 7 or 14 days. The cells were then fixed with 4% paraformaldehyde for 10 min and stained with ALP (Wako). The mineralized bone matrix was stained with 2% ARS after fixation with ethanol.

### Calcein double-labeling

8–9-week-old male mice were intraperitoneally injected with calcein (25 mg/kg, C0875, Sigma-Aldrich) twice at intervals of 3 days. Femurs and tibias of the mice were harvested 4 days after the last injection, fixed with 10% formalin, and dehydrated in a graded series of ethanol. The tissues were then embedded in plastic (Osteo-Bed Bone Embedding kit, Sigma-Aldrich) and sectioned into 7 μm-thick slices. The mineral apposition rate (MAR), mineralizing surface (MS/BS), and bone formation rate (BFR/BS) of femur were analyzed under a confocal microscope.

### LPS-induced calvarial bone destruction

An animal model of LPS-induced bone loss was generated as previously described.^72^ Briefly, PBS or LPS (12.5 mg/kg) was injected into the subcutaneous tissue overlying calvaria of 5–6-week-old male mice. The calvaria were harvested 5 days after the initial injection, fixed, and decalcified, and stained with TRAP.

### OVX-induced postmenopausal osteoporosis

OVX or sham surgery was conducted in 10–11-week-old female mice. Femurs and tibias of the mice were harvested 7 weeks after the surgery and subjected to micro-CT scanning and TRAP staining.

### Statistical analysis

Student’s two-tailed *t*-test or one-way or two-way ANOVA was performed using Prism 8.0.2 (GraphPad) as indicated in the figure legends. *P* < 0.05 was considered statistically significant.

### Supplementary information

Supplementary information

## Data Availability

This study includes no data deposited in external repositories.
