# Supplementary material for: RUFY4 deletion prevents pathological bone loss by blocking endo-lysosomal trafficking of osteoclasts
Source: Bone Res. 2024 May 15;12:29. doi: 10.1038/s41413-024-00326-8 (PMC11094054; doi:10.1038/s41413-024-00326-8)
Supplement: Supplementary file 1 — Supplementary information [file 41413_2024_326_MOESM1_ESM.pdf]

Supplementary information for:

RUFY4 deletion prevents pathological bone loss by blocking endo-lysosomal trafficking of osteoclasts

Running title: RUFY4, a therapeutic target for bone-loss diseases

Minhee Kim[1†], Jin Hee Park[1,2†], Miyeon Go[1], Nawon Lee[1], Jeongin Seo[1], Hana Lee[3], Doyong Kim[3], Hyunil Ha[4], Taesoo Kim[4], Myeong Seon Jeong[5], Suree Kim[6], Taesoo Kim[1,2,7], Han Sung Kim[3], Dongmin Kang[1,6], Hyunbo Shim[1] and Soo Young Lee[1,2,7*]

[1]Department of Life Science, Ewha Womans University, Seoul, 03760, South Korea

[2]The Research Center for Cellular Homeostasis, Ewha Womans University, Seoul, 03760, South Korea

[3]Department of Biomedical Engineering, Yonsei University, Wonju, 26493, South Korea

[4]KM Convergence Research Division, Korea Institute of Oriental Medicine, Daejeon, 34054, South Korea

[5]Chuncheon Center, Korea Basic Science Institute, Chuncheon, 24341, South Korea

[6]Fluorescence Core Imaging Center and Bioimaging Data Curation Center, Ewha Womans University, Seoul, 03760, South Korea

[7]Multitasking Macrophage Research Center, Ewha Womans University, Seoul, 03760, South Korea

[*] Corresponding author. Email: leesy@ewha.ac.kr

[†] These authors contributed equally to this work.

Content

Supplementary Figures 1-11

Supplementary Tables 1-5

Supplementary Figures

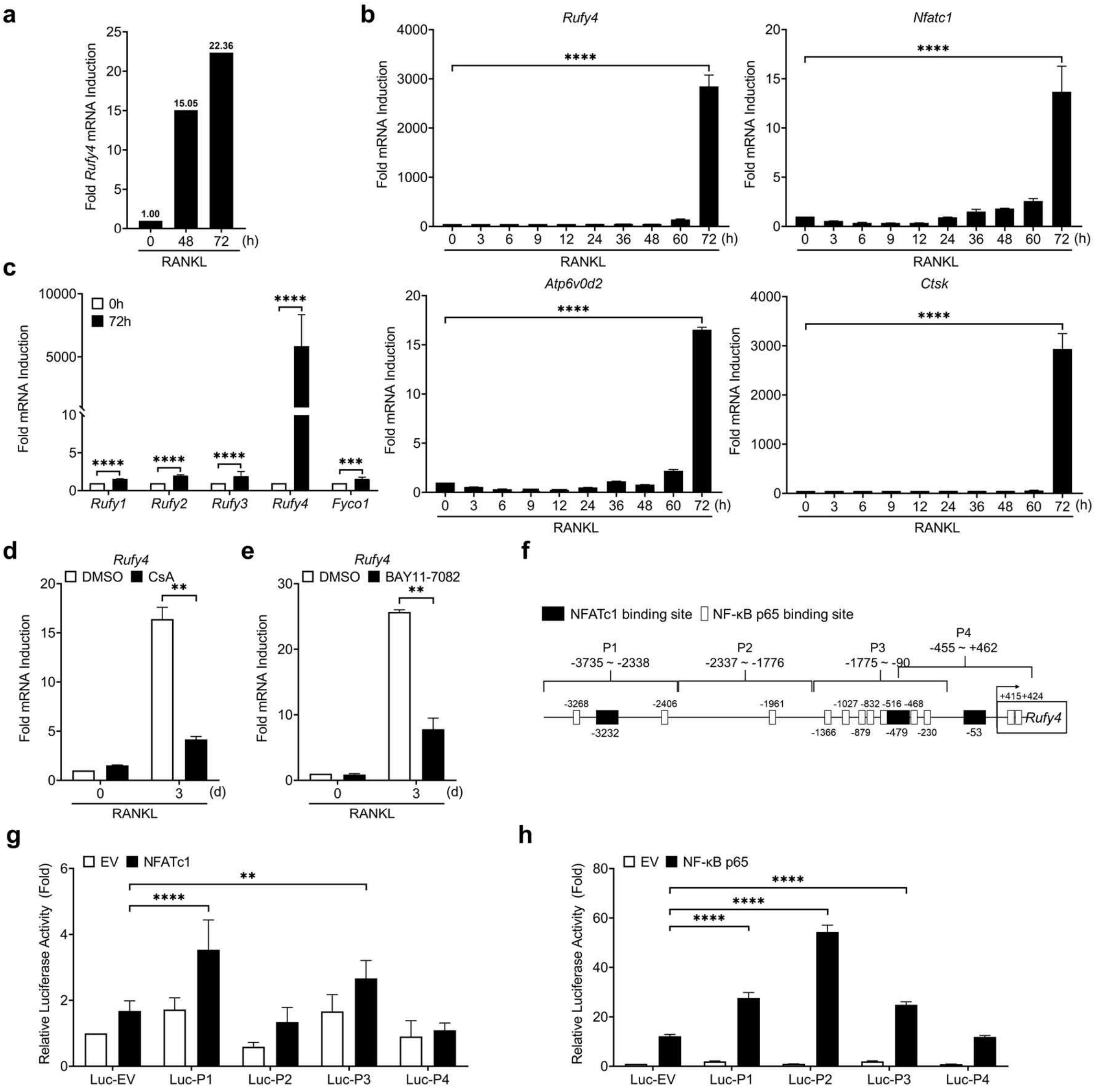

Supplementary Figure 1. RANKL strongly induces *Rufy4* expression in BMMs, and this effect is mediated by NFATc1 and NF-κB p65. a GeneChip analysis of *Rufy4* mRNA levels in wild-type BMMs after they were stimulated with or without RANKL. **b, c** qRT-PCR analysis of the mRNA levels of *Rufy4, Nfatc1, Atp6v0d2* and *Ctsk* (**b**) and the *Rufy* family members (*Rufy1, Rufy2, Rufy3, Rufy4* and *Fyco1*) (**c**) during RANKL-induced osteoclast differentiation. Values are normalized to 0 h level in each member of *Rufy* family. **d, e** qRT-PCR analysis of the *Rufy4* mRNA levels in RANKL-stimulated BMMs that were treated with

cyclosporin A (CsA) (10 μM) (**d**) or BAY11-7082 (5 μM) (**e**). **f** Schematic depiction of the putative NFATc1 and NF-κB p65 binding sites in the promoter region of the *Rufy4* gene. The region upstream of the *Rufy4* transcription initiation sites was divided into four parts, each of which was subcloned into pGL3-Basic luciferase vector (Luc-EV) to generate the Luc-P1 to Luc-P4 *Rufy4* promoter constructs. **g, h** To conduct luciferase-reporter assays, HEK293T cells were co-transfected with Luc-EV or the indicated promoter constructs plus the pcDNA-EV (white) or NFATc1 (**g**) or NF-κB p65 (**h**) expression vector (black). The data are presented as mean ± SD of at least three independent experiments. One-way ANOVA with Tukey's multiple comparison post hoc test (**b**), two-tailed unpaired Student's t test (**c**), or two-way ANOVA with Tukey's multiple comparison post hoc test (**d-e, g-h**) was used for statistical analysis. $^{**}P < 0.01$; $^{***}P < 0.001$; $^{****}P < 0.0001$.

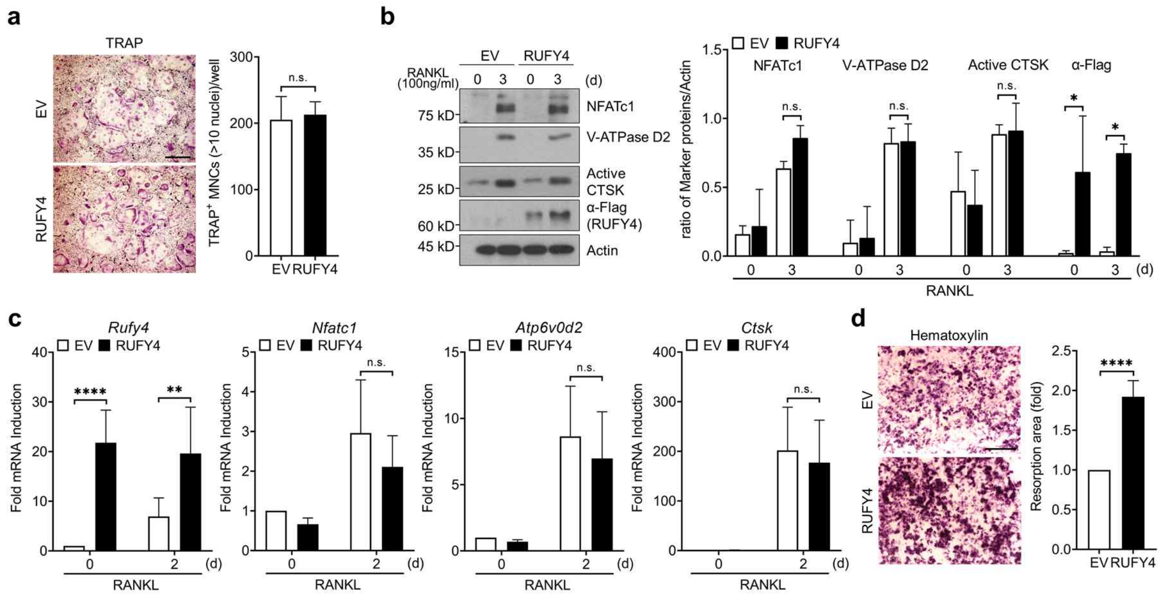

Supplementary Figure 2. RUFY4 overexpression in BMMs has no effect on osteoclast formation but increases their bone resorption. Wild-type BMMs that were transduced with EV or RUFY4 were cultured with M-CSF and RANKL on a plastic surface. **a** The cells were fixed and stained with TRAP. TRAP-positive multinucleated cells (MNCs) were counted (n = 3). Scale bar, 500 μm. **b** Immunoblot analysis of NFATc1, V-ATPase D2 and active CTSK levels in the cell lysates. The band intensities were determined with ImageJ and normalized to actin (n = 3). Actin served as a loading control. Western blots were probed with the indicated antibodies. **c** qRT-PCR analysis of *Rufy4*, *Nfatc1*, *Atp6v0d2* and *Ctsk* mRNA. Assays were performed in duplicate. **d** Wild-type BMMs that were transduced with EV or RUFY4 were cultured with M-CSF and RANKL on bone slices. Hematoxylin staining was conducted to visualize the resorption pit area (n = 4). Scale bar, 250 μm. The data are presented as mean ± SD. Two-tailed unpaired Student's t test (**a, d**) or two-way ANOVA with Tukey's multiple comparison post hoc test (**b, c**) was used for statistical analysis. *$P < 0.05$; **$P < 0.01$; ****$P < 0.0001$; n.s., not significant.

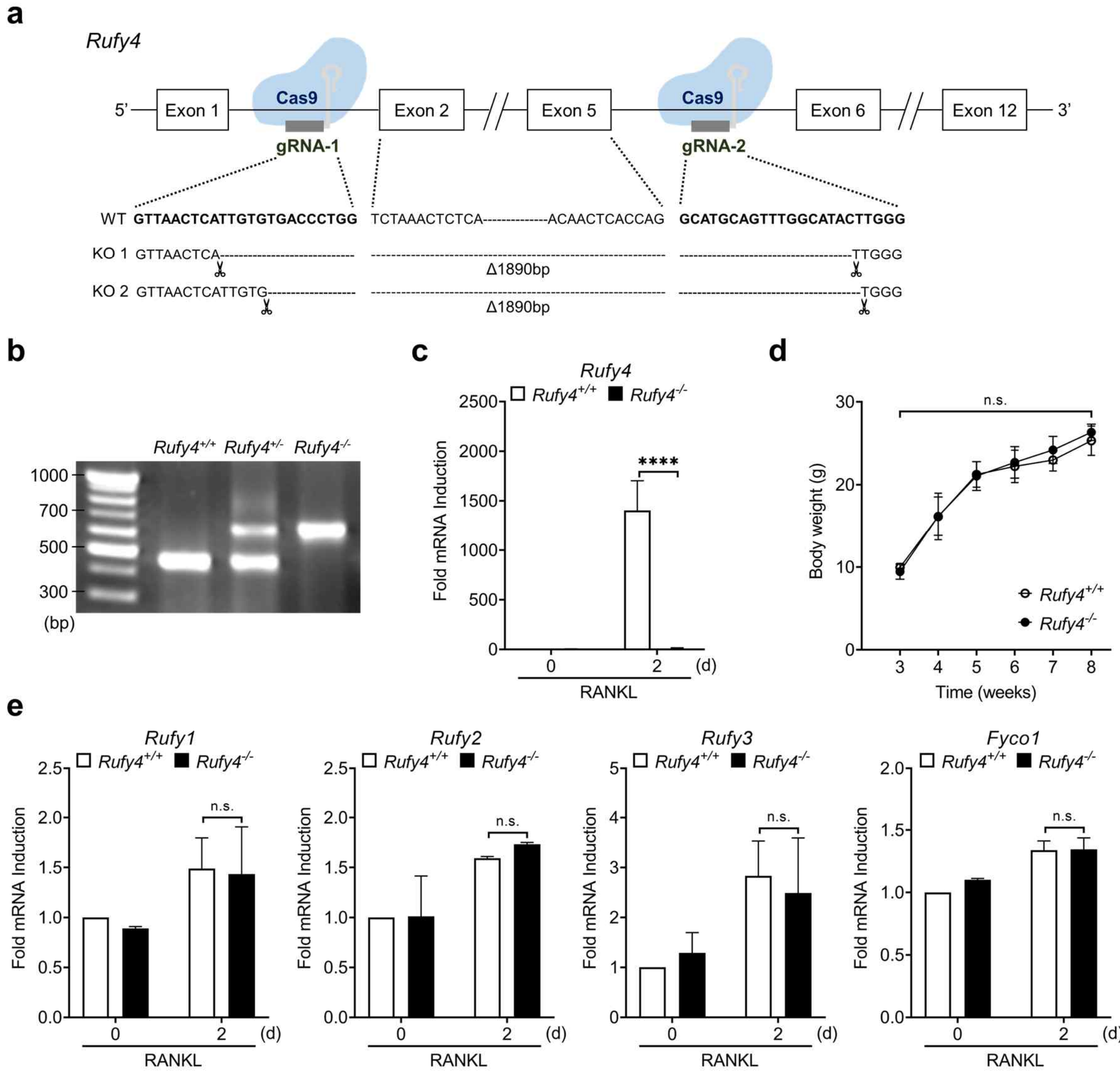

Supplementary Figure 3. Generation and characterization of *Rufy4* global knockout mice. a Schematic depiction of the CRISPR/Cas9-mediated *Rufy4* gene deletion. **b** PCR analysis of *Rufy4*$^{+/+}$, *Rufy4*$^{+/-}$, and *Rufy4*$^{-/-}$ mice. **c** qRT-PCR analysis of the *Rufy4* mRNA in *Rufy4*$^{+/+}$ and *Rufy4*$^{-/-}$ osteoclasts generated from BMMs with M-CSF and RANKL. **d** Body-weight changes in *Rufy4*$^{+/+}$ and *Rufy4*$^{-/-}$ mice (n = 7 mice per group). **e** qRT-PCR analysis of *Rufy1*, *Rufy2*, *Rufy3* and *Fyco1* mRNA in *Rufy4*$^{+/+}$ and *Rufy4*$^{-/-}$ osteoclasts generated from BMMs with M-CSF and RANKL. The data are presented as mean ± SD. Two-way ANOVA with Tukey's multiple comparison post hoc test (**c, e**) or two-tailed unpaired Student's t test (**d**) was used for statistical analysis. ****$P < 0.0001$; n.s., not significant.

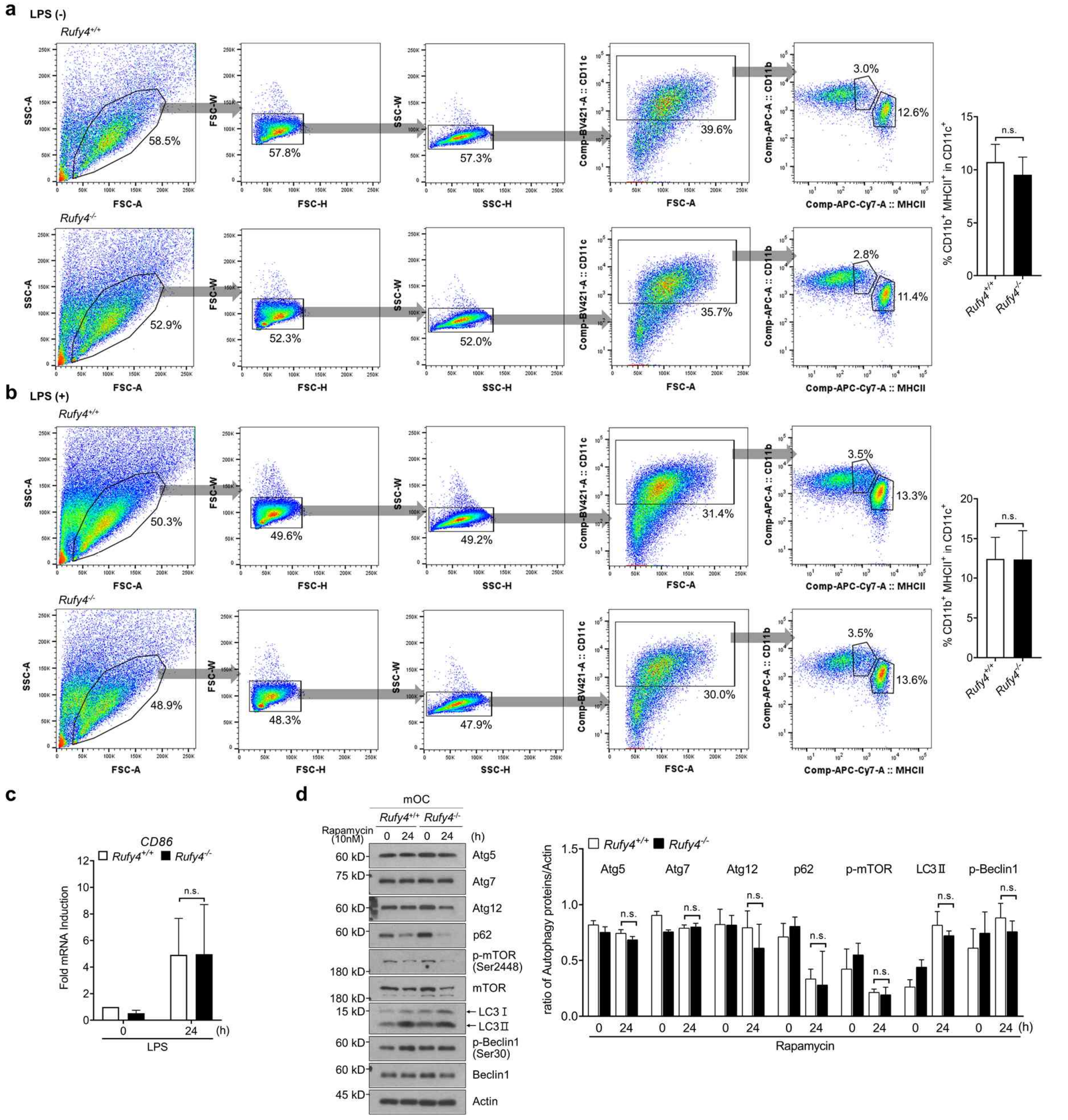

Supplementary Figure 4. *Rufy4*$^{+/+}$ and *Rufy4*$^{-/-}$ mice do not differ in BMDC population or autophagy signaling. a, b Gating strategy for detection of BMDCs after *Rufy4*$^{+/+}$ and *Rufy4*$^{-/-}$ bone marrow cells were treated without (**a**) or with (**b**) LPS. Representative flow cytometry plots of mature BMDCs (CD11c^{+} CD11b^{+} MHCII^{+}) are shown and the mature BMDC frequencies are plotted (n = 3). **c** qRT-PCR analysis of the *cd86* mRNA in *Rufy4*$^{+/+}$ and *Rufy4*$^{-/-}$ BMDCs generated from bone marrow cells with LPS. **d** Immunoblot analysis of the expression and activation of the autophagy signaling molecules Atg5, Atg7, Atg12, p62,

p-mTOR, mTOR, LC3B, p-Beclin1, and Beclin1 in mature *Rufy4*$^{+/+}$ and *Rufy4*$^{-/-}$ osteoclasts that were treated with or without the autophagy inducer, rapamycin (10 nM). The band intensities were determined with ImageJ and normalized to actin (n = 3). Actin served as a loading control. Western blots were probed with the indicated antibodies. The data are presented as mean ± SD. Two-tailed unpaired Student's t test (**a, b**) or two-way ANOVA with Tukey's multiple comparison post hoc test (**c, d**) was used for statistical analysis. n.s., not significant.

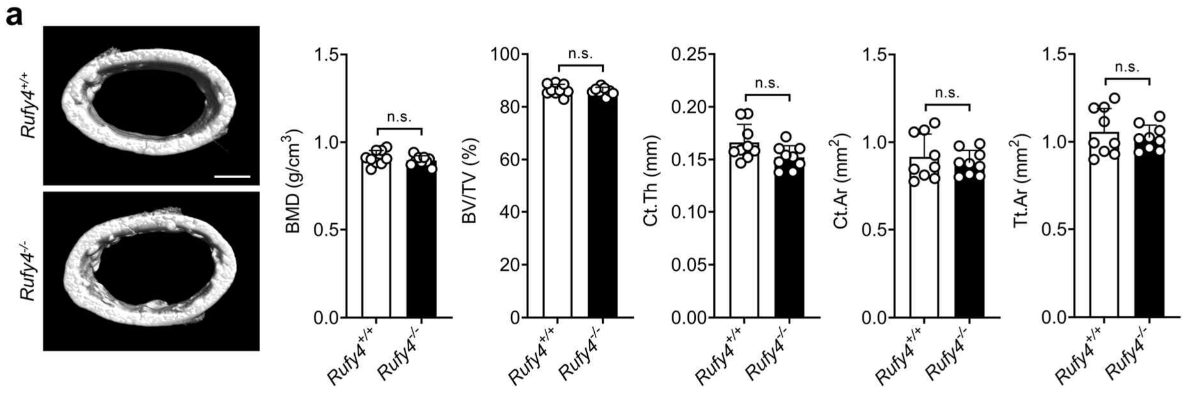

Supplementary Figure 5. *Rufy4*$^{+/+}$ and *Rufy4*$^{-/-}$ mice do not differ in cortical bone phenotype. a Representative micro-CT images of the cortical bone from 8-week-old male *Rufy4*$^{+/+}$ and *Rufy4*$^{-/-}$ mice. Quantification of BMD, BV/TV, Cb.Th, Ct.Ar and Tt.Ar in the cortical bone (n = 9 mice per group). Scale bar, 500 μm. The data are presented as mean ± SD. Two-tailed unpaired Student's *t* test was used for statistical analysis. n.s., not significant.

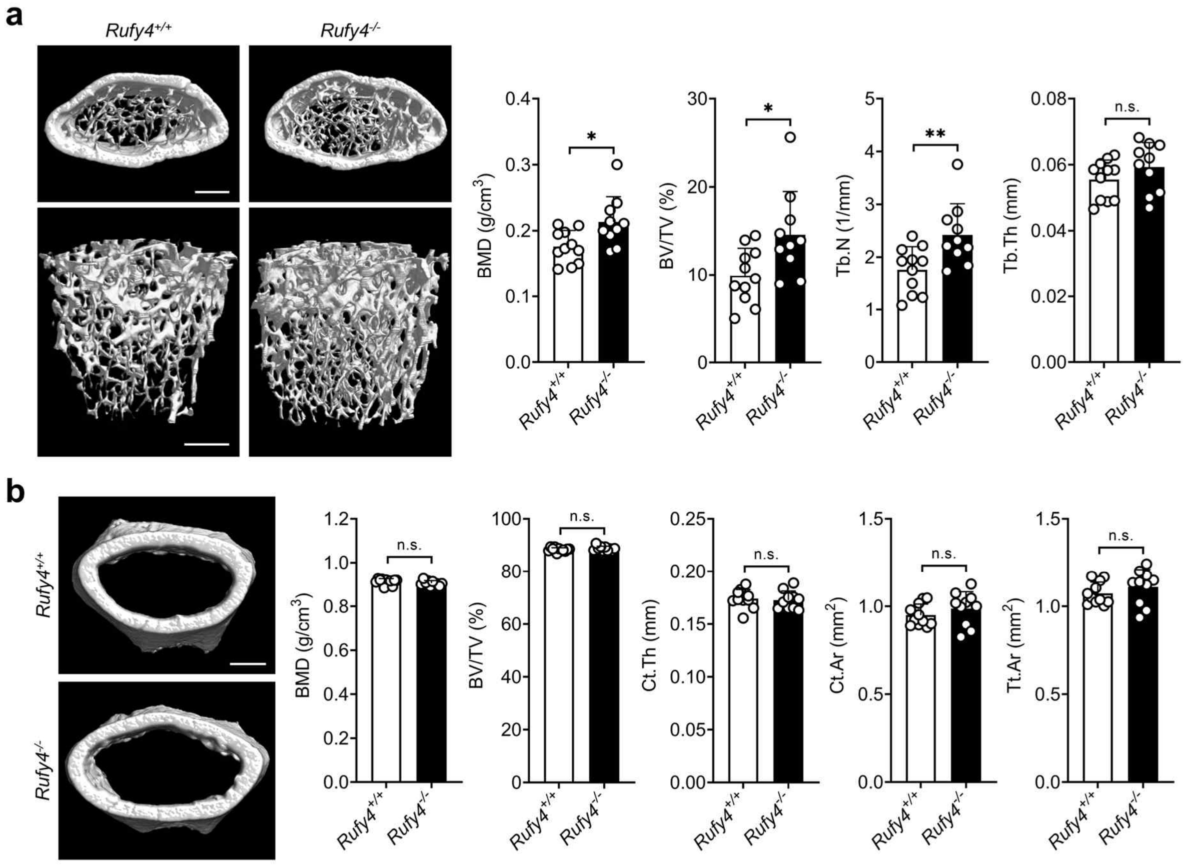

Supplementary Figure 6. Increased trabecular bone mass in 6-month-old *Rufy4*$^{-/-}$ mice. **a** Representative micro-CT images of the distal femur from 6-month-old male *Rufy4*$^{+/+}$ and *Rufy4*$^{-/-}$ mice. Quantification of BMD, BV/TV, Tb.N, and Tb.Th in the distal femur and proximal tibia (n = 10-11 mice per group). **b** Representative micro-CT images of the cortical bone from 6-month-old male *Rufy4*$^{+/+}$ and *Rufy4*$^{-/-}$ mice. Quantification of BMD, BV/TV, Cb.Th, Ct.Ar and Tt.Ar in the cortical bone (n = 10-11 mice per group). Scale bar, 500 μm. The data are presented as mean ± SD. Two-tailed unpaired Student's t test was used for statistical analysis. *$P < 0.05$; **$P < 0.01$; n.s., not significant.

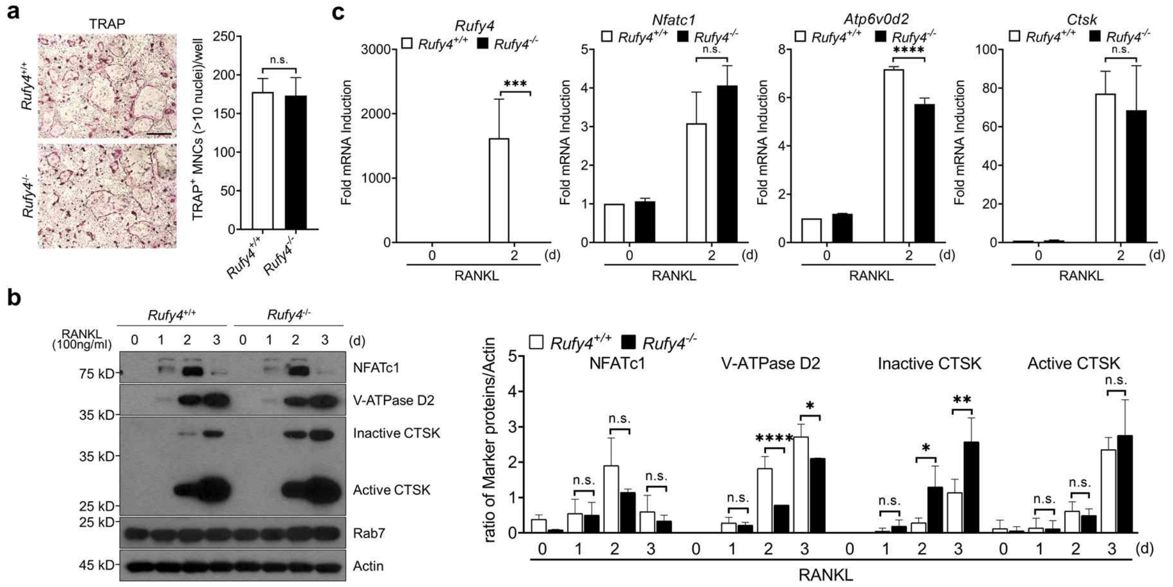

Supplementary Figure 7. ***Rufy4*$^{+/+}$ and *Rufy4*$^{-/-}$ mice do not differ in osteoclast formation.** BMMs from 6-8-week-old male *Rufy4*$^{+/+}$ and *Rufy4*$^{-/-}$ mice were cultured with M-CSF and RANKL on a plastic surface. **a** The cells were stained with TRAP and TRAP-positive MNCs were counted (n = 3). Scale bar, 500 μm. **b** Immunoblot analysis of NFATc1, V-ATPase D2, inactive CTSK, and active CTSK levels in the cell lysates. The band intensities were determined with ImageJ and normalized to actin (n = 3). Actin served as a loading control. Western blots were probed with the indicated antibodies. **c** qRT-PCR analysis of *Rufy4*, *Nfatc1*, *Atp6v0d2* and *Ctsk* mRNA in *Rufy4*$^{+/+}$ and *Rufy4*$^{-/-}$ osteoclasts generated from BMMs with M-CSF and RANKL. The data are presented as mean ± SD of at least three independent experiments. Two-tailed unpaired Student's t test (**a**) or two-way ANOVA with Tukey's multiple comparison post hoc test (**b, c**) was used for statistical analysis. *$P < 0.05$; **$P < 0.01$; ***$P < 0.001$; ****$P < 0.0001$; n.s., not significant.

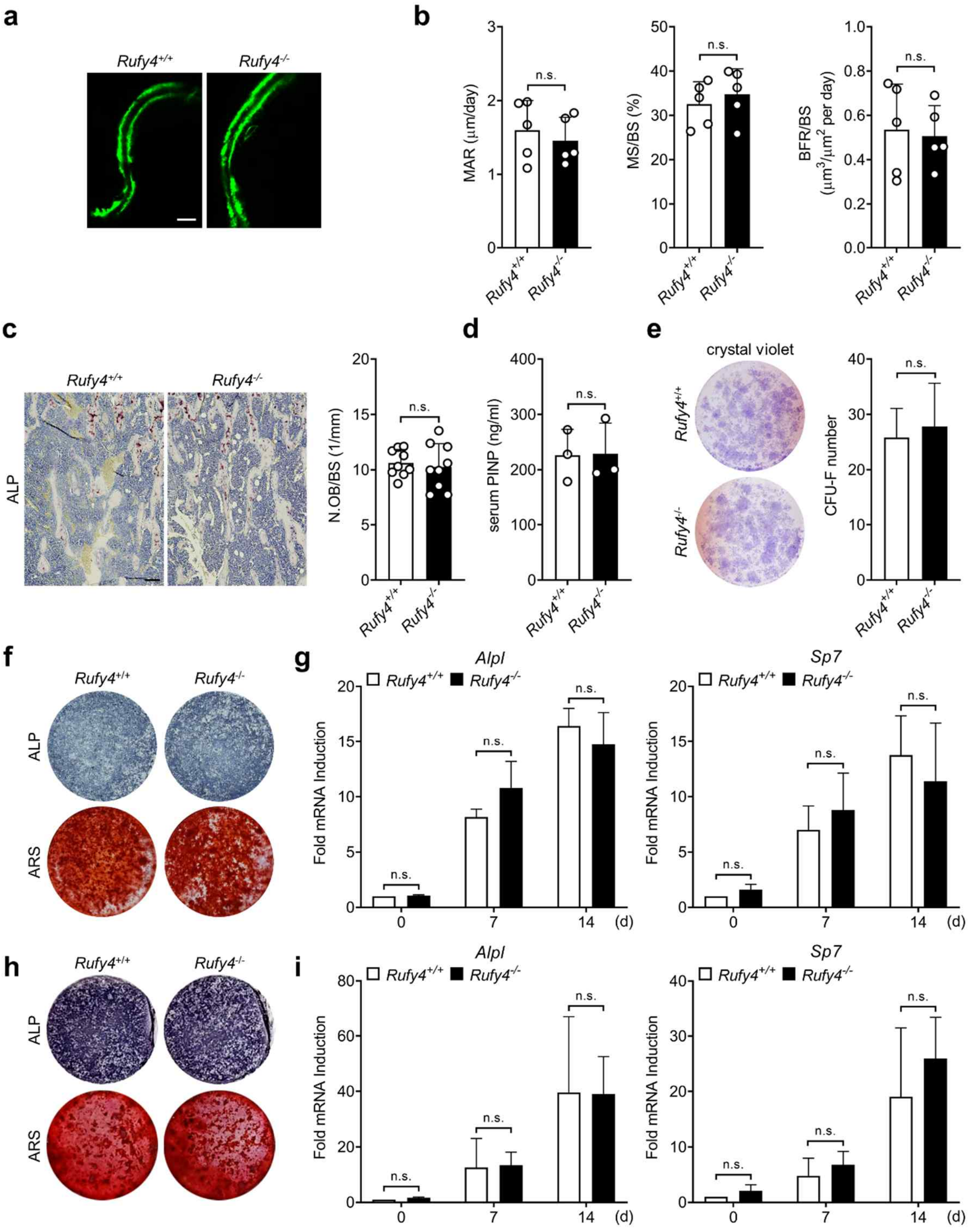

Supplementary Figure 8. *Rufy4*$^{+/+}$ and *Rufy4*$^{-/-}$ mice do not differ in osteoblast activity and differentiation. a Fluorescence images of calcein-labeled trabecular bone from *Rufy4*$^{+/+}$ and *Rufy4*$^{-/-}$ mice. Scale bar, 10 μm. **b** Quantification of mineral apposition rate (MAR), mineralizing surface (MS/BS), and bone formation rate (BFR/BS) (n = 5 mice per group). **c**

Representative alkaline phosphatase (ALP) staining images of the femurs in Fig. 1a. Quantification of osteoblast number per bone surface (N.OB/BS) (*n* = 9 mice per group). **d** PINP protein levels in the serum (*n* = 3 mice per group). **e** Representative crystal violet staining images and quantification of CFU-F number in *Rufy4*$^{+/+}$ and *Rufy4*$^{-/-}$ BMSCs. **f, h** Representative ALP or Alizarin Red S (ARS) staining images of BMSCs-derived (**f**) or calvaria-derived (**h**) osteoblasts from *Rufy4*$^{+/+}$ and *Rufy4*$^{-/-}$ mice (*n* = 3). Original magnification, x1. **g, i** qRT-PCR analysis of *Alpl* and *Sp7* mRNA in BMSCs-derived (**g**) or calvaria-derived (**i**) osteoblasts. Assays were performed in triplicate. The data are presented as mean ± SD of at least three independent experiments. Two-tailed unpaired Student's *t* test (**b-e**) or two-way ANOVA with Tukey's multiple comparison post hoc test (**g, i**) was used for statistical analysis. n.s., not significant.

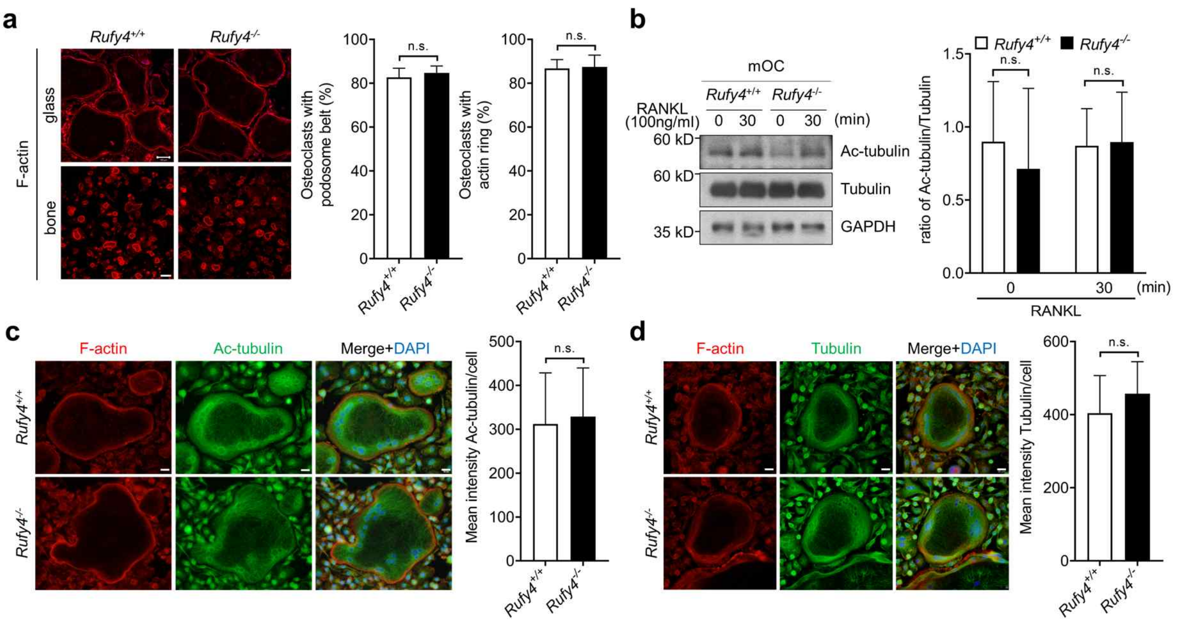

Supplementary Figure 9. *Rufy4*$^{+/+}$ and *Rufy4*$^{-/-}$ osteoclasts do not differ in cytoskeleton organization. a Immunofluorescence staining of filamentous actin (F-actin) in *Rufy4*$^{+/+}$ and *Rufy4*$^{-/-}$ osteoclasts on a glass or bone slices. The frequency of osteoclasts with a podosome belt or actin ring was quantified (n = 3-5). Scale bars, 100 μm (top) and 20 μm (bottom). **b** Immunoblot analysis of acetylated tubulin (Ac-tubulin) and tubulin in *Rufy4*$^{+/+}$ and *Rufy4*$^{-/-}$ osteoclasts on a plastic surface. Ac-tubulin intensity was determined with ImageJ and normalized to tubulin (n = 3). GAPDH served as a loading control. Western blots were probed with the indicated antibodies. **c, d** Immunofluorescence staining of Ac-tubulin (**c**) or Tubulin (**d**) in *Rufy4*$^{+/+}$ and *Rufy4*$^{-/-}$ osteoclasts on a glass. Mean intensity per cell was quantified (n = 3). Scale bars, 20 μm. The data are presented as mean ± SD. Two-tailed unpaired Student's *t* test (**a, c, d**) or two-way ANOVA with Tukey's multiple comparison post hoc test (**b**) was used for statistical analysis. n.s., not significant.

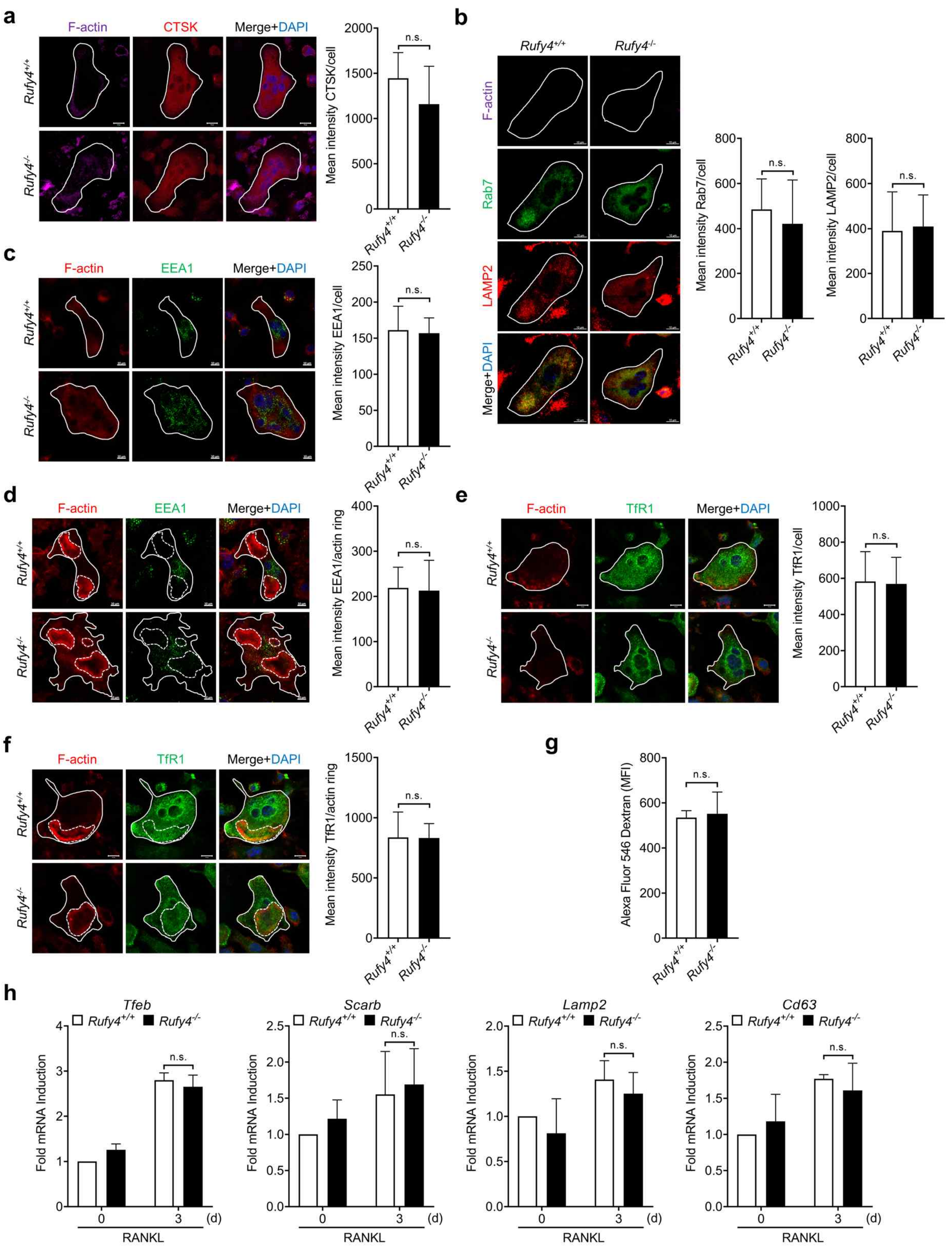

Supplementary Figure 10. ***Rufy4*$^{+/+}$ and *Rufy4*$^{-/-}$ osteoclasts do not differ in the distribution of early and recycling endosomes or the expression of lysosomal markers.**

a, b Immunofluorescence staining of CTSK (**a**) or Rab7 and LAMP2 (**b**) in osteoclasts at

the perinuclear level (upper level expression as described in Fig. 2f). Mean intensity per cell was quantified (*n* = 3). Scale bars, 10 μm. **c, d** Immunofluorescence staining of early endosome antigen 1 (EEA1) in osteoclasts at the perinuclear (**c**) or bone surface level (**d**). Mean intensity per cell or actin ring was quantified (*n* = 3). Scale bars, 10 μm. **e, f** Immunofluorescence staining of transferrin receptor (TfR1) in osteoclasts at the perinuclear (**e**) or bone surface level (**f**). Mean intensity per cell or actin ring was quantified (*n* = 3). Scale bars, 10 μm. **g** MFI of Alexa Fluor 546 dextran in mature *Rufy4*$^{+/+}$ and *Rufy4*$^{-/-}$ osteoclasts (*n* = 6). **h** qRT-PCR analysis of *Tfeb*, *Scarb*, *Lamp2* and *Cd63* mRNA in *Rufy4*$^{+/+}$ and *Rufy4*$^{-/-}$ osteoclasts. The data are presented as mean ± SD of at least three independent experiments. Two-tailed unpaired Student's *t* test (**a-g**) or two-way ANOVA with Tukey's multiple comparison post hoc test (**h**) was used for statistical analysis. n.s., not significant.

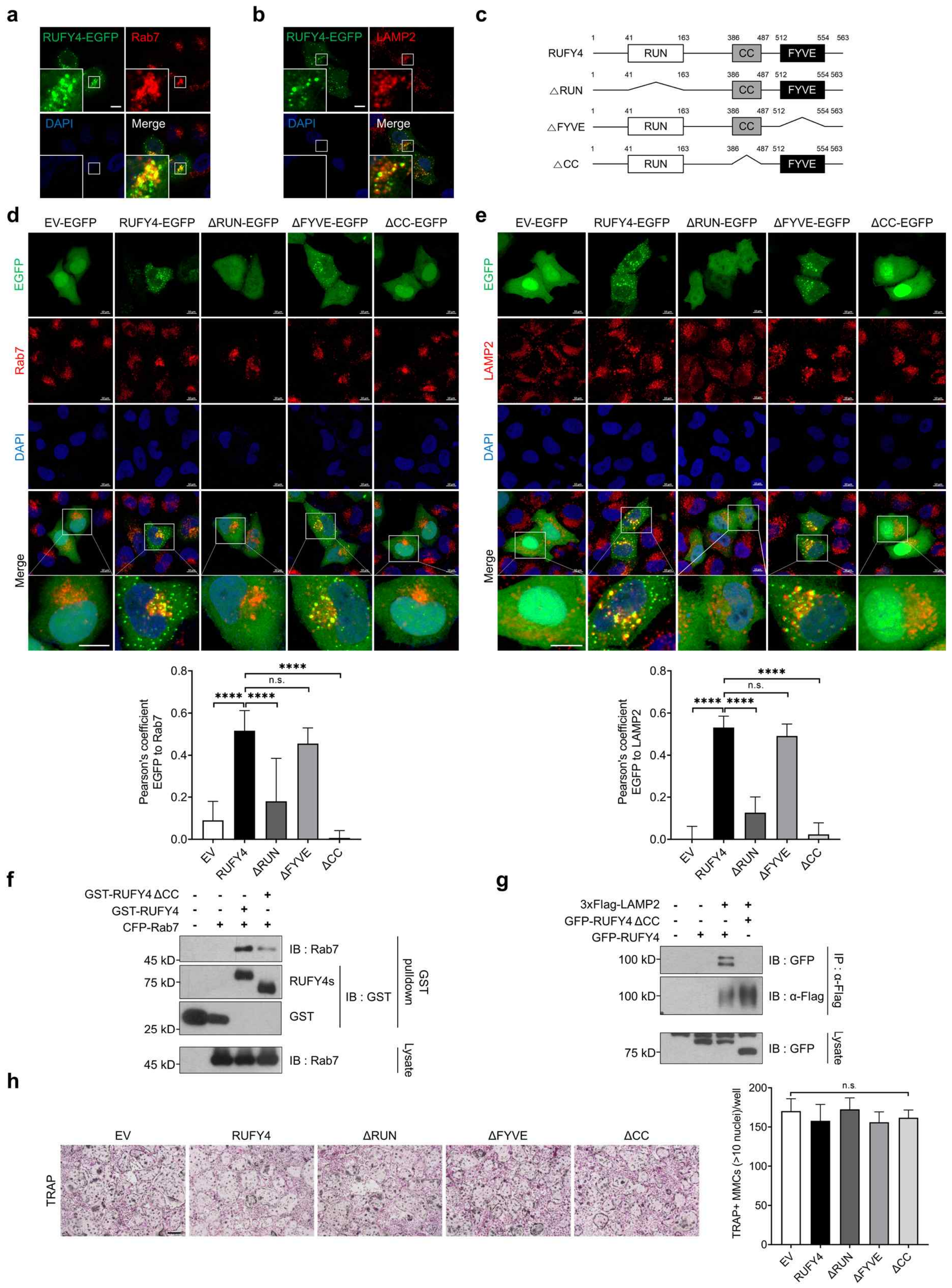

Supplementary Figure 11. All RUFY4 domains are required for interaction with endo-lysosomal proteins and bone resorption but not for osteoclast differentiation. a, b

Immunofluorescence staining of Rab7 (**a**) or LAMP2 (**b**) in HeLa cells that were transfected with RUFY4-EGFP. Scale bar, 10 μm. **c** Schematic depiction of the RUFY4 deletion mutants. **d, e** Immunofluorescence staining of Rab7 (**d**) or LAMP2 (**e**) in HeLa cells that were transfected with EV-EGFP, RUFY4-EGFP, or the ΔRUN-EGFP, ΔFYVE-EGFP, or ΔCC-EGFP mutants. Pearson's correlation coefficient is a statistic for quantifying the co-localization of each construct with Rab7 or LAMP2 (n = 3). Scale bars, 10 μm. **f, g** HEK293T cells were transfected with the ΔCC mutant of RUFY4 and subjected to co-IP analyses of its association with Rab7 (**f**) or LAMP2 (**g**) (n = 3). Western blots were probed with the indicated antibodies. **h** BMMs from *Rufy4*$^{-/-}$ mice were transduced with EV, RUFY4, or the ΔRUN, ΔFYVE, or ΔCC mutants, treated with M-CSF and RANKL on a plastic surface. The cells were then stained with TRAP and TRAP-positive MNCs were counted (n = 3). Scale bar, 500 μm. The data are presented as mean ± SD. One-way ANOVA with Tukey's multiple comparison post hoc test was used for statistical analysis. ****P < 0.0001; n.s., not significant.

Supplementary Tables

Table S1. List of antibodies for co-IP and western blots

Name	Host species	Source	Cat no.	Dilutions
**Cathepsin K**	mouse monoclonal	Santa Cruz	sc-48353	1:1000
**Rab7**	rabbit monoclonal	Cell signaling	9367S	1:1000
**Actin**	mouse monoclonal	Santa Cruz	sc-47778	1:1000
**α-Flag**	mouse monoclonal	Sigma-Aldrich	F3165-.2MG	1:2500
**GST**	mouse monoclonal	Santa Cruz	sc-138	1:1000
**GFP**	mouse monoclonal	Santa Cruz	sc-9996	1:1000
**LAMP2**	rabbit polyclonal	Invitrogen	PA1-655	1:1000
**NFATc1**	mouse monoclonal	Santa Cruz	sc-7294X	1:1000
**V-ATPase D2**	rabbit monoclonal	provided by Y. Choi (University of Pennsylvania, PA)		1:5000
**Atg5**	rabbit monoclonal	Cell signaling	12994S	1:1000
**Atg7**	rabbit monoclonal	Cell signaling	8558S	1:1000
**Atg12**	rabbit monoclonal	Cell signaling	4180S	1:1000
**p62**	rabbit polyclonal	Invitrogen	PA5-20839	1:1000
**p-mTOR**	rabbit monoclonal	Cell signaling	5536S	1:1000
**mTOR**	mouse monoclonal	Cell signaling	4517	1:1000
**LC3**	rabbit monoclonal	Cell signaling	2775S	1:1000
**p-Beclin1**	rabbit monoclonal	Cell signaling	35955S	1:1000
**Beclin1**	rabbit monoclonal	Cell signaling	3495S	1:1000
**Ac-tubulin**	mouse monoclonal	Sigma-Aldrich	T6793	1:1000
**Tubulin**	rat monoclonal	Abcam	ab6160	1:1000
**GAPDH**	mouse monoclonal	Santa Cruz	sc-32233	1:1000

Table S2. List of antibodies for immunofluorescence staining

Name	Host species	Source	Cat no.	Dilutions
**Cathepsin K**	mouse monoclonal	Santa Cruz	sc-48353	1:500
**Rab7**	rabbit monoclonal	Abcam	ab137029	1:200
**LAMP2 (mouse)**	rat monoclonal	Developmental Studies Hybridoma Bank	GL2A7	1:200
**LAMP2 (human)**	mouse monoclonal	Developmental Studies Hybridoma Bank	H4B4	1:100
**Ac-tubulin**	mouse monoclonal	Sigma-Aldrich	T6793	1:100
**Tubulin**	rat monoclonal	Abcam	ab6160	1:100
**EEA1**	mouse monoclonal	BD Biosciences	610456	1:200
**Transferrin receptor (H68.4)**	mouse monoclonal	Life Technologies	13-6800	1:500
**phalloidin-iFluor 594**		Abcam	ab176757	1:1000
**phalloidin-iFluor 647**		Abcam	ab176759	1:1000

Table S3. List of antibodies for flow cytometry

Name	Host species	Source	Cat no.	Dilutions
**CD16/CD32**	mouse monoclonal	BD Biosciences	553141	1:1000
**CD11b APC**	rat monoclonal	BioLegend	101211	1:1000
**CD11c Brilliant violet 421**	Hamster monoclonal	BioLegend	117329	1:1000
**CD86 Alexa Fluor 488**	rat monoclonal	BioLegend	105017	1:1000
**CD40 PE**	rat monoclonal	BioLegend	124609	1:1000
**APC/Cyanine7**	rat monoclonal	BioLegend	107627	1:1000

Table S4. List of primers for cloning (Enzyme sites are in bold.)

Name	Forward (5' - 3')	Reverse (5' - 3')
**3xFlag-CMV-RUFY4**	**EcoR1-** GAA**GAATTC**ATGGCCAACAACGGGACC	**Kpn1-** GAAT**GGTACC**GAGGTGTCCTGGATTTCTTC
**3xFlag-CMV-ΔRUN**	**EcoR1-** GAA**GAATTC**ACCATGCAGCGGCCAGACCTGGATGAA	**Kpn1-** GAAT**GGTACC**GAGGTGTCCTGGATTTCTTC
**3xFlag-CMV-ΔFYVE**	**EcoR1-** GAA**GAATTC**ATGGCCAACAACGGGACC	**Kpn1-** GAAT**GGTACC**GATCCAGGGGCCACAGTAGA
**3xFlag-CMV-ΔCC**	**EcoR1-** GAA**GAATTC**ATGGCCAACAACGGGACC<br>**Middle F-** GAGAAGCCACAAGAGGTGTTGGAACTGATCCAT	**Middle R-** ATGGATCAGTTCCAACACCTCTTGTGGCTTCTC<br>**Kpn1-** GAAT**GGTACC**GAGGTGTCCTGGATTTCTTC
**pMX-3xFlag-RUFY4**	**EcoR1-** GAA**GAATTC**ATGGCCAACAACGGGACC	**Xho1-** GAA**CTCGAG**TCACTACTTGTCATCGTCATCCTTGTA
**pMX-3xFlag-ΔRUN**	**EcoR1-** GAA**GAATTC**ACCATGCAGCGGCCAGACCTGGATGAA	**Xho1-** GAA**CTCGAG**TCACTACTTGTCATCGTCATCCTTGTA
**pMX-3xFlag-ΔFYVE**	**EcoR1-** GAA**GAATTC**ATGGCCAACAACGGGACC	**Xho1-** GAA**CTCGAG**TCACTACTTGTCATCGTCATCCTTGTA
**pMX-3xFlag-ΔCC**	**EcoR1-** GAA**GAATTC**ATGGCCAACAACGGGACC	**Xho1-** GAA**CTCGAG**TCACTACTTGTCATCGTCATCCTTGTA
**pEBG-RUFY4**	**Spe1-** GAA**ACTAGT**ATGGCCAACAACGGGACCATC	**Not1-** GAA**GCGGCCGC**TCAGGTGTCCTGGATTTCTTC
**pEBG-ΔRUN**	**Spe1-** GAA**ACTAGT**ACCATGCAGCGGCCAGACCTGGATGAA	**Not1-** GAA**GCGGCCGC**TCAGGTGTCCTGGATTTCTTC

**pEBG-ΔFYVE**	**Spe1-** GAA**ACTAGT**ATGGCCAACAACGGGACCATC	**Not1-** GAA**GCGGCCGC**TCATCCAGGGGCCACAGTAGA
**pEBG-ΔCC**	**Spe1-** GAA**ACTAGT**ATGGCCAACAACGGGACCATC	**Not1-** GAA**GCGGCCGC**TCAGGTGTCCTGGATTTCTTC
**EGFP-N3-RUFY4**	**EcoR1-** GAA**GAATTC**ATGGCCAACAACGGGACC	**Sal1-** GAA**GTCGAC**GGTGTCCTGGATTTCTTC
**EGFP-N3-ΔRUN**	**EcoR1-** GAA**GAATTC**ACCATGCAGCGGCCAGACCTGGATGAA	**Sal1-** GAA**GTCGAC**GGTGTCCTGGATTTCTTC
**EGFP-N3-ΔFYVE**	**EcoR1-** GAA**GAATTC**ATGGCCAACAACGGGACC	**Sal1-** GAA**GTCGAC**TCCAGGGGCCACAGTAGA
**EGFP-N3-ΔCC**	**EcoR1-** GAA**GAATTC**ATGGCCAACAACGGGACC	**Sal1-** GAA**GTCGAC**GGTGTCCTGGATTTCTTC
**3xFlag-CMV-LAMP2**	**Not1-** GAA**GCGGCCGC**ATGTGCCTCTCTCCGGTTAAAGG	**Kpn1-** GAAT**GGTACC**GACAGAGTCTGATATCCAGCATAGG
**pGL3-P1-luc**	**Nhe1-** GAA**GCTAGC**AGGTAACCTGCAT	**Hind3-** GAA**AAGCTT**CCACTAGGGCACA
**pGL3-P2-luc**	**Nhe1-** GAA**GCTAGC**TGTAGCCAGTCAA	**Hind3-** GAA**AAGCTT**CTGCCCTCCTGCG
**pGL3-P3-luc**	**Nhe1-** GAA**GCTAGC**AGGCTCGAGCGCC	**Hind3-** GAA**AAGCTT**AGGGGGCTGGACC
**pGL3-P4-luc**	**Nhe1-** GAA**GCTAGC**CAGGGCTGGAAGG	**Hind3-** GAA**AAGCTT**CTGGCATGATCCT

Table S5. List of primers for qRT-PCR

Name	Forward (5' - 3 ′)	Reverse (5' - 3 ′)
mActin	AGATGTGGATCAGCAAGCAG	GCGCAAGTTAGGTTTTGTCA
mRufy4	AGCAGAAGCCTGAAAAATGCAG	CCGTCGAAGGGCAGTGAATA
mNfatc1	CCAGAAAATAACATGCGAGCC	GTGGGATGTGAACTCGGAAG
mAtp6v0d2	CAGAGATGGAAGCTGTCAACATTG	TGCCAAATGAGTTCAGAGTG
mCtsk	GGAAGAAGACTCACCAGAAGC	GCTATATAGCCGCCTCCACAG
mCd86	CATGGGCTTGGCAATCCTTA	AAATGGGCACGGCAGATATG
mRufy1	CTCTGTCTGAAGGACGCACA	TCGTCTTTTCCAAGCCATCT
mRufy2	TCCACTCATTCCACCTTGCC G	GGATACGGGTCTGTTGGGAG
mRufy3	TATGCCAACCCCCACCAC	TCTGTCAAATCCTCCCAAGAG
mFyco1	TTCCTTTTCCCTCTCAAGCA	GCCACATGGATCGTGAATTA
mAlpl	GCTGATCATTCCCACGTTTT	CTGGGCCTGGTAGTTGTTGT
mSp7	TGAGGAAGAAGCCCATTCAC	ACTTCTTCTCCCGGGTGTG
mTfeb	CCACCCCAGCCATCAACAC	CCACCCCAGCCATCAACAC
mScarb	AGAAGGCGGTAGACCAGAC	GTAGGGGGATTTCTCCTTGGA
mLamp2	TGTATTTGGCTAATGGCTCAGC	TATGGGCACAAGGAAGTTGTC
mCd63	GAAGCAGGCCATTACCCATGA	TGACTTCACCTGGTCTCTAAACA
